# Somatic Mutations in Oncogenes Are in Chronic Myeloid Leukemia Acquired *De Novo via* Deregulated Base-Excision Repair and Alternative Non-Homologous End Joining

**DOI:** 10.3389/fonc.2021.744373

**Published:** 2021-09-20

**Authors:** Nikola Curik, Vaclava Polivkova, Pavel Burda, Jitka Koblihova, Adam Laznicka, Tomas Kalina, Veronika Kanderova, Jana Brezinova, Sarka Ransdorfova, Dominika Karasova, Katerina Rejlova, Marina Bakardjieva, Daniela Kuzilkova, David Kundrat, Jana Linhartova, Hana Klamova, Cyril Salek, Pavel Klener, Ondrej Hrusak, Katerina Machova Polakova

**Affiliations:** ^1^Institute of Hematology and Blood Transfusion, Prague, Czechia; ^2^Institute of Pathological Physiology, First Faculty of Medicine, Charles University, Prague, Czechia; ^3^Second Faculty of Medicine, Charles University, Prague, Czechia; ^4^CLIP-Childhood Leukaemia Investigation Prague, Department of Paediatric Haematology and Oncology, Second Faculty of Medicine, Charles University and Motol University Hospital, Prague, Czechia; ^5^First Department of Internal Medicine-Department of Hematology, Charles University General Hospital in Prague, Prague, Czechia

**Keywords:** chronic myelogenous leukemia (CML), base excision repair (BER), alt-nonhomologous end joining (alt-NHEJ), TKI resistance, oncogene

## Abstract

Somatic mutations are a common molecular mechanism through which chronic myeloid leukemia (CML) cells acquire resistance to tyrosine kinase inhibitors (TKIs) therapy. While most of the mutations in the kinase domain of BCR-ABL1 can be successfully managed, the recurrent somatic mutations in other genes may be therapeutically challenging. Despite the major clinical relevance of mutation-associated resistance in CML, the mechanisms underlying mutation acquisition in TKI-treated leukemic cells are not well understood. This work demonstrated *de novo* acquisition of mutations on isolated single-cell sorted CML clones growing in the presence of imatinib. The acquisition of mutations was associated with the significantly increased expression of the *LIG1* and *PARP1* genes involved in the error-prone alternative nonhomologous end-joining pathway, leading to genomic instability, and increased expression of the *UNG*, *FEN* and *POLD3* genes involved in the base-excision repair (long patch) pathway, allowing point mutagenesis. This work showed *in vitro* and *in vivo* that *de novo* acquisition of resistance-associated mutations in oncogenes is the prevalent method of somatic mutation development in CML under TKIs treatment.

## Introduction

Tyrosine kinase inhibitors (TKIs) are effective for treating chronic myeloid leukemia (CML); however, 10-15% of patients develop resistance to first line imatinib treatment.^1^ The 1^st^ generation TKI imatinib remains the most prescribed drug recommended for frontline therapy of chronic phase (CP) CML. While 2^nd^-generation TKI frontline therapy has shown faster cytogenetic and molecular responses, no differences in overall survival were found between 2^nd^ generation TKIs and imatinib, which may be the preferred choice for older patients with comorbidities and for most adult patients with low- and intermediate risk CML-CP (1, 2).

Mutations in the kinase domain (KD) of BCR-ABL1 represent a common molecular mechanism of imatinib therapy resistance and are responsible for 30% of acquired imatinib resistance cases (3, 4). Furthermore, CML relapses and progression to advanced phases of the disease are often associated with recurrent mutations in oncogenes, which are frequently mutated in other hematological malignancies (5, 6). Despite the major clinical relevance of mutation-associated TKI resistance in CML, the mechanisms underlying mutation acquisition in oncogenes of drug-resistant CML cells are not yet well understood.

It was postulated, that BCR-ABL1 KD mutations could be detected in newly diagnosed patients in accelerated or blast phases (7). This led to the general assumption that a preexisting mutated subpopulation of CML cells carrying a resistance phenotype is selected and expanded during TKI treatment. However, some studies observed the mechanism of *de novo* BCR-ABL1 mutation acquisition during imatinib treatment in CML cells. It was suggested that BCR-ABL1 expression, but not kinase activity, is required for the acquisition of KD mutations (8). The NAD^+^-dependent histone deacetylase SIRT1 was shown to promote acquisition of BCR-ABL1 KD mutations in CML cells (9). Others proposed the activity of the alternative nonhomologous end-joining (alt-NHEJ) pathway in resistant CML cells driven by BCR-ABL1 and/or MYC transcriptional activity (10, 11).

This study was based on the hypothesis that the appearance of somatic mutations during imatinib treatment is not just a passive process of selection and expansion of preexisting mutated clones; rather, it is based on *de novo* mutation acquisition involving deregulation of the DNA damage response and DNA repair pathways.

## Materials and Methods

The detailed methods are provided as **Supplementary Methods**.

### CML Cell Lines

CML cell lines KCL-22 (ACC 519) and CML-T1 (ACC 7) were obtained from a publicly accessible biological resource center (Leibniz Institute - Deutsche Sammlung von Mikroorganismen und Zellkulturen GmbH/DSMZ, Braunschweig, Germany).

### Isolation of CML Clones

KCL-22 and CML-T1 clones were prepared by single-cell FACS sorting (BD FACS Aria III; BD Biosciences, San Jose, CA, USA) according to CD38 expression ([HB-7] anti-CD38 APC; Sony Biotechnology, San Jose, CA, USA) in 96-well plates containing optimal growing medium either imatinib-free (sensitive; S clones) or with a 0.004 µM imatinib that was subsequently increased up to 4 µM (resistant; R clones) (**Supplementary Figure S1**).

### Patients

CML patients (n=32) were diagnosed and treated at the Institute of Hematology and Blood Transfusion in Prague (UHKT), Czech Republic (**Tables 1**, **2**). All samples were collected after written informed consent was obtained according to the principles of the Declaration of Helsinki and approval by the UHKT Ethics Committee.

**Table 1:** CML patients characteristics who failed to TKI with detected BCR-ABL1 mutations **(A)** BCR-ABL1 mutation detection at the time of therapy failure.

A									
Patient No.	Age at diagnosis	Disease status at diagnosis	Total follow-up (months)	Months from diagnosis	Months on TKI therapy	TKI at the time of mutation detection	Line of TKI	Type of BCR-ABL1 mutation	
1	52	CP	22.7	22.7	22.7	IM	1	F359V 8%. Y253H 5%	
2	40	CP	60.3	45.1	44.7	NILO	2	F317L 54%	
3	39	CP	67.4	4.8	4.2	IM	1	G250E 10%. M244V 1%	
4	35	CP	78.3	60.4	60.3	IM	1	V379I 100%	
5	33	CP	112.6	79.7	72.2	DASA	4	M437V 58%	
6	58	CP	110.1	8.1	7.4	IM	1	M351T 78%. G250E 22%	
7	64	CP	68.2	13.7	12.7	IM	1	M244V 3%	
8	38	CP	99.4	28.1	28.1	IM	1	S417Y 82%	
9	55	CP	120.4	79.7	79.6	IM	1	M244V 100%	
10	66	CP	51.5	20.4	20.2	NILO	2	V299A 23%	
11	55	CP	29.1	15.3	15.3	IM	1	Y253C 28%	
12	47	CP	42.1	11.7	11.6	IM	1	F317S 5%	
13	71	CP	96.8	66.1	66.1	IM	1	G250E 80%. Y253H 20%	
14	69	CP	11.7	4.5	3.2	IM	1	T315I 100%	
15	68	CP	60.4	9.5	9.5	IM	1	E255K 5%	
16	51	CP	58.2	55.7	55.4	IM	1	F311L 6%	
17	47	CP	121.1	118.2	117.4	IM	1	F317L 2%. E255V 1%	
18	73	CP	25.8	7.2	7.2	IM	1	F359V 100%	
19	56	AP	16.6	12.5	10.7	IM	1	Q252H 100%	
20	80	CP	43.8	9.3	8.8	IM	1	T315I 100%	
21	76	CP	11.8	11.8	11.8	IM	1	G250E 16%	
22	66	CP	45.8	12.2	6.7	NILO	1	Y253H 70%	
23	20	CP	46.9	6.9	4.2	IM	1	E255V 76%. E255K 6%	
24	25	CP	78.0	8.5	8.0	IM	1	T315I 1%	
**B**									
**Patient No.**	**Age at diagnosis**	**Disease status at diagnosis**	**BCR-ABL1 IS %**	**Months from diagnosis**	**Months on TKI therapy**	**TKI at the time of mutation detection**	**Line of TKI**	**Mutations in oncogenes**	
								**Detected at the time of therapy failure**	**Detected at the time of diagnosis**
1	52	CP	0.2	22.7	22.7	IM	1		
2	40	CP	21.0	45.1	44.7	NILO	2	ASXL1 R693X 11%	
3	39	CP	27.0	4.8	4.2	IM	2		
4	35	CP	0.3	63.6	63.5	IM	1		
5	33	CP	0.2	79.7	79.5	DASA	4		
6	58	CP	2.8	12.4	11.7	IM	1	ASXL1 H630Hfs 9%	ASXL1 H630Hfs 32%
7	64	CP	0.1	13.7	12.7	IM	1	SETD2 Q1343X 1%	SETD2 Q1343X 41%
8	38	CP	0.3	28.1	27.6	IM	1		
9	55	CP	0.1	79.6	79.6	IM	1		
10	66	CP	0.3	20.4	20.2	NILO	2		
11	55	CP	4.2	15.3	15.1	IM	1	ASXL1 Q977X 2%	ASXL1 Q977X 34%
12	47	CP	0.2	11.7	11.6	IM	1		
13	71	CP	0.1	66.1	66.1	IM	1		
14	69	CP	93.0	4.5	3.2	IM	1		
15	68	CP	19.0	37.2	36.3	DASA	4	ASXL1 W796X 9%	ASXL1 W796X 3%
16	51	CP	0.4	55.7	55.5	IM	1		
17	47	CP	0.2	118.2	117.4	IM	1		
18	73	CP	29.0	12.9	12.5	DASA	2	WT1 C453Y 29%	
19	56	AP	13.0	12.5	9.8	IM	1		
20	80	CP	38.0	23.6	23.1	NILO	2		
21	76	CP	6.5	11.8	11.8	IM	1		
22	66	CP	28.0	49.4	44.0	IM	3	ASXL1 G645delinsWfs 36%	ASXL1 G645delinsWfs 9%
23	20	CP	8.1	6.9	4.2	IM	1		
24	25	CP	76.0	8.5	8.0	DASA	2	RUNX1 D198N 14%	

BCR-ABL1 mutations were not detected at the time of diagnosis in none of 21/24 patients. In 3 patients, analysis at the time of diagnosis could not be done due to lack of archived samples (pts. no. 6, 7 and 11). **(A)** BCR-ABL1 mutation detection at the time of therapy failure. **(B)** Mutation detection in other oncogenes at the time of therapy failure. The presence of mutations in oncogenes was retrospectively analyzed at the time of diagnosis in all 24 patients with available DNA samples. CP, chronic phase; AP, accelerated phase; IM, imatinib; NILO, nilotinib; DASA, dasatinib.

**Table 2:** Characteristics of patients and samples used for the evaluation of gene expression analysis of DNA repair and response to DNA damage genes.

Patient No	Diagnosis	Sex	Age	EUTOS score	ELTS score	Type of sample	Time of analysis (in month)/response on imatinib	BCR-ABL1% IS	Mutation status	Folow up/2020
23	23.06.2016	F	21	low	low	BM CD34+ cells	4M/no optimal	8.1	BCR-ABL1 E255V 76%, E255K 6%	DMR after HSCT
24	20.09.2013	M	25	high	high	PB	Diagnosis	62	wt	CML related death
						PB	8M/no optimal	76	BCR-ABL1 T315I 1% RUNX1 D198N 13.6%	
25	08.11.2016	M	73	low	intermediate	BM CD34+ cells	Diagnosis	34	wt	CML not related death
						BM CD34+ cells	3M/optimal	0.51	wt	
26	19.10.2016	M	33	low	intermediate	BM CD34+ cells	Diagnosis	37	wt	DMR on 2^nd^ line TKI (dasatinib)
						BM CD34+ cells	3M/no optimal	19	wt	
27	13.02.2017	F	69	low	intermediate	BM CD34+ cells	12M/optimal	0.076	–	MMR (0.015% BCR-ABL1) on 1^st^ line TKI (imatinib)
28	02.01.2018	M	46	low	low	BM CD34+ cells	3M/no optimal	23	wt	MMR on 2^nd^ line TKI (dasatinib)
29	13.03.2012	M	39	low	low	BM CD34+ cells	Diagnosis	75	wt	DMR on 2^nd^ line TKI (dasatinib)
30	27.09.2016	F	50	low	low	BM CD34+ cells	10M/optimal	0.0089% MR4	–	DMR on 1^st^ line TKI (imatinib)
31	04.04.2019	M	70	low	intermediate	PB	Diagnosis	70	wt	CML related death
						BM CD34+ cells	Diagnosis	70	wt	
						BM CD34+ cells	5M/no optimal	15	BCR-ABL1 T315I 100%	
32	31.05.2007	F	60	low	high	PB	Diagnosis	395	wt	CML related death
						PB	6M/no optimal	26	BCR-ABL1 G250E 1%	

PB, peripheral blood; BM, bone marrow; WT, wild type; DMR, deep molecular response; MMR, major molecular response.

### *In Vivo* Models

Experiments were performed on NOD.Cg-*Prkdc^scid^* *Il2rg^tm1Wjl^*/SzJ (NSG)-immunodeficient female mice (n=16) aged 8-10 weeks. Mice were subcutaneously inoculated with 1x 10^6^ KCL-22R imatinib-resistant clones. Treated mice were orally administered imatinib (100 mg/kg/mouse) once per day, with the initiation of treatment 3 days after inoculation. The design of all experiments was approved by the institutional Animal Care and the Use Committee.

### Droplet Digital PCR

Mutations were analyzed on DNA level using commercially available allele-specific assays for droplet digital PCR (ASO-ddPCR) (QX200 Droplet Digital PCR system and Auto Droplet generator, Bio-Rad, Hercules, CA, USA).

### Gene Expression PCR Arrays

Human DNA Damage Signaling Pathway RT^2^ Profiler™ PCR Array (PAHS-029Z; Qiagen, Valencia, CA, USA) and Human DNA Repair RT^2^ Profiler™ PCR Array (PAHS-042Z; Qiagen) were performed on the StepOnePlus system (Thermo Fisher Scientific, Waltham, MA, USA). The complete list of 128 measured genes is provided (**Supplementary Table S1**). The differentially expressed genes were determined following stringent criteria: 1) the mean 2^ΔC^ value for R clones was ≥ 2.5 or ≤ 0.4-fold of the mean 2-(Cts-Ctc) value for S clones and 2) the difference of 2^ΔC^ values for R clones and S clones reached the level of significance p=0.05.

### Next-Generation Sequencing

BCR-ABL1 KD amplicon libraries were prepared using the Nextera XT DNA Library Prep Kit (cat. number FC-131-1096, Illumina, San Diego, CA, USA).

The DNA custom-designed NGS panel of 33 genes often mutated in myeloid malignancies (**Supplementary Table S2**) was analyzed using SeqCap EZ HyperCap Workflow (Roche, San Diego, CA, USA).

Sequence analysis and mutation identification were performed using NextGENe software (SoftGenetics, State College, PA, USA).

### Mass Cytometry

The protein expression in leukemic cells and clones was determined by staining with CyTOF metal-conjugated antibodies against proteins of interest (**Supplementary Table S3**) and analyzed by a CyTOF2 mass cytometer (Fluidigm, South San Francisco, CA, USA) as described in detail previously (12).

### Statistical Analysis

The statistical analyses were performed using Student´s t-test in MS Excel (Microsoft Corporation, Redmond, WA, USA). Fish plots were generated in the program Fishplot package for R (13).

## Results

### TKI-Resistant Mutation Acquisition in CML Patients

From 253 CML patients diagnosed within the years 2010-2018 and treated with TKIs as frontline therapy 87 responded to therapy as warning or failure according to the ELN recommendation.^1^ This group of non-optimally responded patients were regularly evaluated for the presence of BCR-ABL1 KD mutations. BCR-ABL1 mutations were detected in 24/87 patients (28%) at a median time of 12.2 months since TKI start (range 3.2-117.4; **Table 1A**). Presence of BCR-ABL1 mutations was retrospectively analyzed by NGS at the time of diagnosis in 21/24 patients with available RNA samples. Mutations were not detected in any of the 21 patients at the time of diagnosis with the sensitivity range of 1-3.7% (**Supplementary Methods**). Thus, it is highly probable, that these mutations were acquired *de novo* under TKI treatment. DNA NGS myeloid panel (**Supplementary Table S2**) was used to reveal co-occurrence of somatic mutations in oncogenes in the samples of non-optimal responders with BCR-ABL1 mutations. The oncogenic mutations were detected in 8/24 patients (33%) (**Table 1B**). Presence of oncogenic mutations was retrospectively analyzed by NGS myeloid panel at the time of diagnosis in all 24 patients. The analysis revealed mutations in 5/24 patients at the time of diagnosis, while 3 mutations were likely acquired *de novo* under TKI treatment.

### Resistance-Conferring Mutations Are Acquired *De Novo* After Exposure to Imatinib

The hypothesis of *de novo* mutation acquisition in CML cells resistant to TKI was tested on FACS sorted single cells of KCL-22 exposed to imatinib. To address whether the putative BCR-ABL1 mutation acquisition is related to CD38 (non)expression as suggested previously (14), cells were sorted according to the expression of CD38. Out of the 120 sorted single cells, 33 growing clones emerged. No BCR-ABL1 KD mutations were detected by NGS in KCL-22 clones in day 21 post-sorting. Finally, 6 KCL-22R clones resistant to 4 µM imatinib were established from both CD38- (n=4) and CD38+ (n=2) cells of origin (**Supplementary figure S1A**).

**Figure 1:**
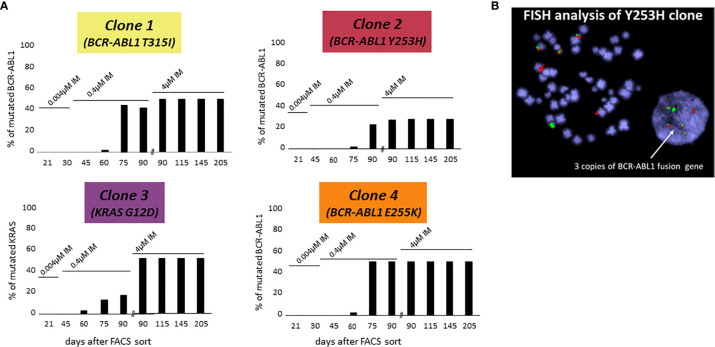
*De novo* acquisition of somatic mutations during the development of KCL-22 clones of CD38- cell of origin resistant to 4 µM imatinib. KCL-22R clones were prepared by single cell FACS sorting into medium with 0.004 µM imatinib. The imatinib concentration was increased to 0.4 µM in day 31 and further increased to 4 µM in day 75 after sort. **(A)** The presence of mutated mRNA for BCR-ABL1 or DNA for KRAS during the development of resistant KCL-22R clones up to day 205 post single-cell FACS sorting: Clone 1, Clone 2, Clone 3 and Clone 4. No BCR-ABL1/KRAS mutations were detected in KCL-22R clones until days 60-75 postscoring after the escalation of the imatinib concentration up to 0.4 µM. The growing concentration of imatinib is indicated above the columns. The presence of mutation in day 90 was determined both in the culture with 4 µM imatinib and in the precedent culture with 0.4 µM imatinib growing simultaneously. **(B)** FISH analysis XL BCR/ABL DF of Clone 2 (Y253H) revealed the presence of 3 BCR-ABL1 fusions.

The following three of four CD38- KCL-22R clones carried a mutation in the BCR-ABL1 KD: Clone 1, T315I (50%); Clone 2, Y253H (30%); and Clone 4, E255K (50%) (**Figure 1A**). DNA analyses using ASO-ddPCR confirmed the same level of mutated genomic BCR-ABL1 in the resistant clones, suggesting that clones with 50% mutated BCR-ABL1 carried mutations on only 1 Ph chromosome. The presence of an additional cryptic unmutated BCR-ABL1 fusion was confirmed in Clone 2 with 30% mutated BCR-ABL1 by FISH analysis (**Figure 1B**).

In Clone 3, BCR-ABL1 was unmutated during the whole follow-up. The NGS myeloid panel was applied to all four KCL-22R clones growing in 4 µM imatinib (**Supplementary Table S4**). The analysis revealed that Clone 3 was bearing mutations in the *ATRX* (K1933Kfs), *RUNX1* (G74R) and *KRAS* (G12D) genes. The *ATRX* and *RUNX1* mutations were first detected 21 days post-sorting. The *KRAS* mutation, confirmed by Sanger sequencing in day 205 in 4 µM imatinib, was first detected 60 days post-sorting on the 0.4 µM imatinib level, similar to the acquisition of BCR-ABL1 mutations in other KCL-22R clones (**Figure 1A**). Y253H-bearing Clone 2 acquired a *de novo* mutation in *BCOR* (R1454Q). CD38+ KCL-22R Clone 5 and Clone 6 acquired BCR-ABL1 mutations Y235H and T315I, respectively (**Supplementary Figure S2**).

**Figure 2:**
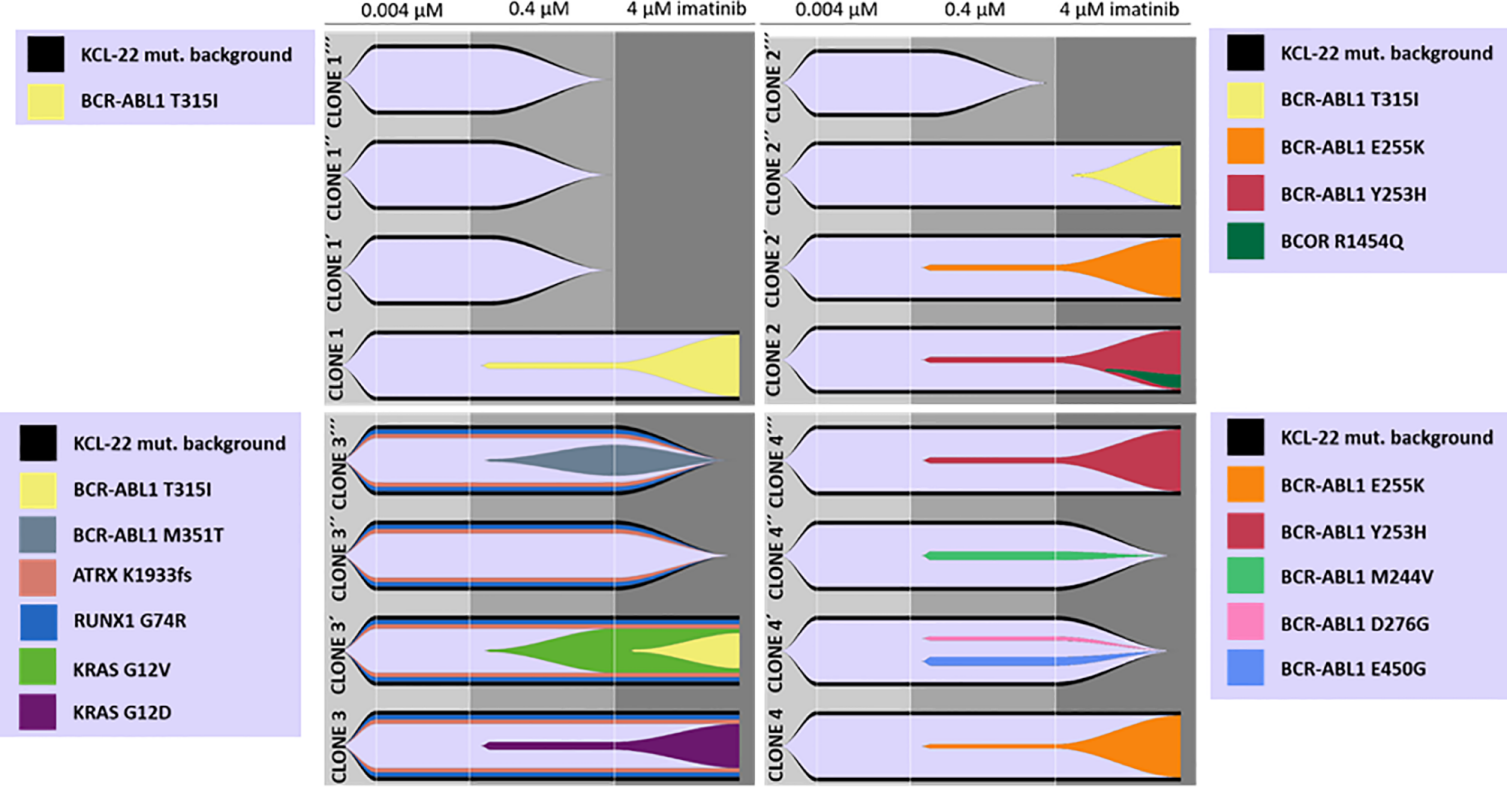
*De novo* acquisition and evolution of somatic mutations during the development of KCL-22 clones resistant to 4 µM imatinib. Originally, 4 single-cell FACS-resistant CD38- clones, Clone 1, Clone 2, Clone 3 and Clone 4, represented by the lower fish plots in each quadrant, were isolated by a stepwise increase in imatinib concentration and analyzed for mutations by a DNA NGS custom panel. The cells of each clone (using cryopreserved samples from day 21 after sorting) were used to repeat resistance development in biological triplicates, represented by ´, ´´ and ´´´ fish plots in each quadrat. The imatinib concentration was gradually increased up to 4 µM during repeated resistance development, performed exactly as before. DNA NGS custom panel analysis revealed newly obtained resistant clones that exhibit different spectra of acquired mutations, while some clones failed to repeatedly gain a resistant phenotype. The Fishplot package for R was used for data visualization. KCL-22 mutation background is shown elsewhere in detail (**Supplementary Table S4**).

To support data suggesting *de novo* acquisition of mutations conferring imatinib resistance, cells of the original isolated KCL-22 Clones 1-4 stored at day 20 after the sort to 0.004µM IM were used to repeat experiments of resistance development in biological triplicates. Interestingly, the newly isolated resistant clones (KCL-22R´) developed a different spectrum of BCR-ABL1 and *KRAS* mutations (**Figure 2**).

The *de novo* acquisition of mutations was also confirmed in the experiment with CML-T1 cell line by similarly designed experiment (**Supplementary Results**; **Supplementary Figure S3**). KCL-22 and CML-T1 cells have capability of mutation acquisition irrespectively to the expression of CD38.

### *De Novo* Acquisition of Mutations in CML Cells Is Associated With Transcriptional Activation of the Base-Excision Repair and Alt-NHEJ Repair Pathways *In Vitro* and *In Vivo*

Based on the observed results, the *de novo* acquisition of mutations during imatinib treatment seems to be the result of the interplay between stochastic processes and impaired DNA damage signaling and repair. To test this hypothesis, gene expression PCR arrays carrying DNA damage response and DNA repair genes were applied to reveal the distinct expression profiles of KCL-22R and KCL-22S (sensitive) clones, respectively (**Supplementary Results**; **Supplementary Figure S4A**) and to identify the genes with significantly different expression in KCL-22R clones at the time of mutation acquisition. For this and following analysis, KCL-22R clones originating from CD38- cells were used. Two distal clusters were formed from KCL-22R clones and KCL-22S clones, respectively. Eleven significantly differentially expressed genes were identified: decreased *CDKN1A* and increased *BRCA2*, *BRIP1*, *CDC25A*, *EXO1*, *FEN1*, *H2AFX*, *LIG1*, *MLH1*, *RAD51* and *UNG*. Differentially expressed genes were analyzed with KEGG Pathways (https://www.genome.jp/kegg/pathway.html) and Reactome Pathways (https://reactome.org/) databases to delineate putative dysregulated molecular mechanisms/pathways. Mismatch repair and BER pathways were proposed as the most relevant by KEGG (p<.001 and p=0.0001, respectively), and homology repair and mismatch repair were proposed by the Reactome. However, K-means clustering supported the transcriptional activity of BER (**Supplementary Figures S4B, C** and **Supplementary Results**).

**Figure 3:**
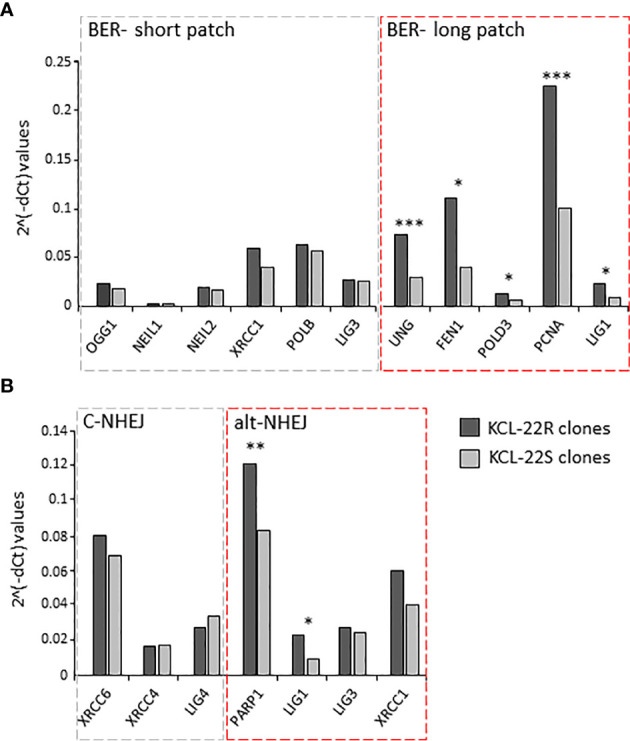
The expression of DNA damage response and DNA repair genes putatively associated with mutagenesis in KCL-22 clones. The expression data are shown for the genes of short- and long-patch BER **(A)** and for DNA ligases and their functional partners - LIG1 and PARP1; LIG3 and XRCC1; LIG4, XRCC4 and XRCC6, respectively, involved in classical/c-NHEJ and alternative/alt-NHEJ **(B)** pathways. The expression was measured at the days of mutation acquisitions; 60-75 days postsorting. Dark gray columns represent the average gene expression for KCL-22R imatinib-resistant clones. Light gray columns represent the average gene expression in control, imatinib-naïve KCL-22S clones. The gene expression was normalized to mean Ct values for 4 control genes (B2M, GAPDH, RPLP0, HPRT1). The level of significance is indicated: *P < 0.05, **P < 0.01 and ***P < 0.001.

To decipher the putative pathway involved in mutation acquisition, the expression of genes associated with mismatch repair (*RPM1*, *RFC1*, *POLD3*, *PCNA*, *LIG1*, *EXO1*, *MLH1*, *MSH6*, *MLH3*, *MSH3* and *PMS2*) was determined in KCL-22R clones and compared to that in KCL-22S clones. The increased expression of *MLH1* and genes with more general functions in DNA repair (e.g., *PCNA*, *POLD3*, *LIG1*) was found, while the expression of genes involved in the direct recognition of mismatched DNA (*MSH6, MLH3, MSH3, PMS2*) was not significantly altered, making mismatch repair a questionable candidate to participate in mutation acquisition in KCL-22R clones (**Supplementary Figure S5A**). Next, the expression of genes participating in BER was determined. The expression of genes involved in short-patch BER was not altered, while significant upregulation of genes participating in long-patch BER was found (**Figure 3A**). In addition to its roles in BER and mismatch repair, *LIG1* is also associated with the error-prone alt-NHEJ pathway of DNA double-strand break (DSB) repair. To investigate the possible role of DNA DSB repair pathways, the expression of DNA ligases and their functional partners was determined. The expression of *LIG1* and *PARP1* was found to be significantly increased, while the expression of *LIG3, LIG4* and their respective partners was not altered (**Figure 3B**). Increased expression of BER and alt-NHEJ genes was also confirmed at the time of mutation acquisition in the imatinib-resistant KCL-22R´ and CML-T1R clones (**Supplementary Figure S5B, C**). Moreover, regulatory regions of the *LIG1* and *PARP1* genes were found to be epigenetically active during and after mutation acquisition and, in the case of *LIG1*, occupied by the MYC transcription factor, while the DNA methylation was found in KCL-22 at regulatory region of *LIG4* (**Supplementary Results**; **Supplementary Figures S6–S8**). PARP1 inhibitor NU-1025 treatment prevented or delayed BCR-ABL1 mutation acquisition in CML-T1 and KCL-22 cells after the exposure to imatinib (**Supplementary Results**; **Supplementary Figure S9**).

**Figure 4:**
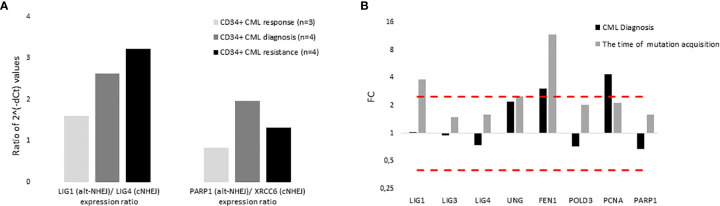
The expression of DNA ligases, their functional partners and BER – long patch genes in primary cells of CML patients. **(A)** The columns represent the ratios of 2-(Cts-Ctc) values of gene expression for indicated pairs of genes (involved either in altNHEJ or cNHEJ pathway) in CD34+ BM cells of CML patients at the time of optimal response to imatinib (n = 3; Nr. 25, Nr. 27, Nr. 30; light gray), at the CML diagnosis (n = 4; Nr. 25, Nr. 26, Nr. 29, Nr. 31; dark gray) and at the time of resistance to imatinib (n = 4; Nr. 23, Nr. 26, Nr. 28, Nr. 31; black). **(B)** The black columns represent the fold change (FC; relative expression) of average gene expression in total PB leukocytes of CML patients at the time of diagnosis (n = 3; Nr. 24, Nr. 31 and Nr. 32) compared to gene expression in healthy donors (n = 5; pooled). The gray columns represent the FC of average gene expression in total PB leukocytes of CML patients (n = 2; Nr. 24 and Nr. 32) at the time of first detection of somatic mutations at low levels (RUNX1 D198N 14% + T315I 1% and BCR-ABL1 G250E 1%, respectively) compared to expression in healthy donors (n = 5; pooled). Red dashed lines mark cut-offs indicating strongly differentially expressed genes with FC ≤ 0.4 or ≥ 2.5, similar to inclusion criteria applied for KCL-22R clones.

The expression of DNA ligases and their functional partners was examined in CD34+ primary cells of CML-CP patients (**Table 2**). The expression of *LIG1* and *PARP1*, representing alt-NHEJ pathway, was found to be relatively augmented to that of *LIG4* and *XRCC6*, representing classical NHEJ (c-NHEJ) pathway, at the time of diagnosis and resistance to imatinib (therapy failure) in comparison with prevalently non-leukemic CD34+ cells at the time of response to TKIs (**Figure 4A**). Gene expression was also examined in the total leukocytes from peripheral blood (PB) of CML patients at diagnosis (n=3), who on TKI therapy subsequently acquired mutations, compared with the pooled samples of PB leukocytes of healthy donors (n=5) (**Figure 4B**). No difference in the expression of DNA ligases was found, while the increased expression of *FEN1*, *PCNA* and, to a lesser degree, *UNG* was confirmed in CML-CP. Suggested molecular mechanisms were studied during mutation acquisition in CML patients resistant to TKIs. The expression of *LIG1*, *FEN1, UNG* and, to a lesser degree, *POLD3* was augmented during mutation acquisition (**Figure 4B**).

### *De Novo* TKI-Resistant Mutation Acquisition and the Evolution of Resistant CML Clones Depends on the Culture Conditions, Time, and Dose of Imatinib

The processes of natural mutation appearance and mutated cell expansion was studied in detail in KCL-22 cells. Invariably, no BCR-ABL1 KD mutations were detected in imatinib-naïve cells by NGS. However, BCR-ABL1 mutations were repeatedly detected by NGS after the exposure of cells to 0.4 µM imatinib. The competition of mutated clones was driven not solely by the type of mutations but also by their frequency and by the environmental conditions (**Supplementary Results**; **Supplementary Figure S10**). In progressed KCL-22 cells (Supplementary Methods), BCR-ABL1 mutations did not appear after the exposure of cells to 0.4 µM imatinib. Compared to *de novo* KCL-22 cells, the progressed cells revealed decreased sensitivity to imatinib and putative involvement of BCR-ABL1-independent resistance mechanisms (**Supplementary Results**; **Supplementary Figure S11**).

**Figure 5:**
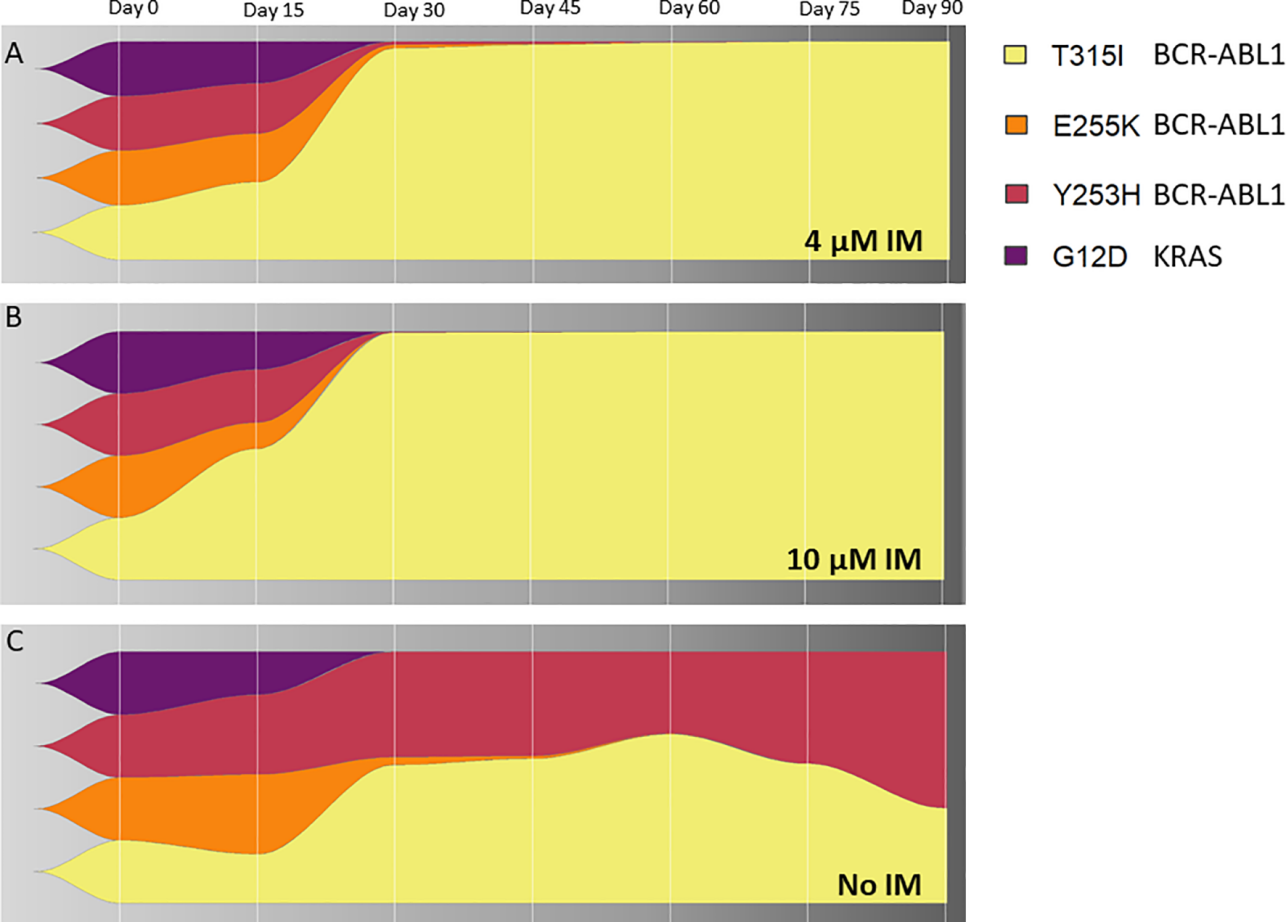
The clonal evolution of KCL-22R resistant clones. Starting from an equal number of cells (1x10^6^) for each clone (Clone 1, Clone 2, Clone 3 and Clone 4 of KCL-22) growing in 4 µM imatinib, subsequent clonal evolution was followed by the monitoring of mutated BCR-ABL1 transcripts and mutated KRAS at selected time points indicated above the charts. Cells were cultivated for 90 days in stable conditions of **(A)** 4 µM imatinib, **(B)** 10 µM imatinib or **(C)** without imatinib. The superior growing fitness of Y253H-bearing Clone 2 in imatinib-free conditions is likely due to the presence of additional unmutated BCR-ABL1 fusion in this clone, as revealed by detected copy numbers by ddPCR and confirmed by cytogenetic analysis. The presence of BCR-ABL1 KD and KRAS mutations is shown as a percentage of total BCR-ABL1 or KRAS in the cells, respectively, determined by ASO-ddPCR.

To address a clonal evolution upon the constant imatinib dose, equal numbers of cells from the 4 KCL-22R clones growing in 4 µM imatinib were mixed, split, and cultured in 4 µM, 10 µM imatinib and imatinib-free medium. The T315I-bearing clone outgrew other 3 clones in both 4 µM and 10 µM imatinib (**Figures 5A, B**). The superior proliferation of the T315I-bearing clone in 4 µM imatinib was also confirmed using a mixture of cell tracking dyes (**Supplementary Figure S12A**) and by cell cycle analysis (**Supplementary Figures S12B, C**). In the mixture of clones growing without imatinib, the Y253H-bearing clone showed a proliferative advantage over the other 3 clones (**Figure 5C**). Clonal evolution was followed for the artificially mixed 3 CML-T1R clones, where Y253H-mutated cells outgrown in all conditions (**Supplementary Figure S13**).

The signaling properties of leukemic cells and resistant clones were studied at the protein level using a CyTOF custom-designed panel. BCR-ABL1-dependent pathways *via* STAT5, ERK1/2 and AKT were inhibited after the imatinib exposure and remained diminished in mutated resistant clones with slightly distinct pathway preferences dependent on the type of mutation and/or imatinib dose. Imatinib-resistant clones KCL-22R displayed persistently high expression of MYC, while overexpression of BCL-2 was additionally found in CML-T1R clones (**Supplementary Results**; **Supplementary Figures S14–S17**).

**Figure 6:**
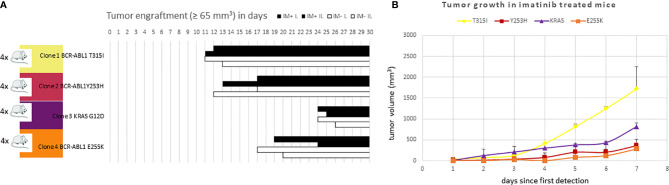
The efficacy of **(A)** engraftment and **(B)** tumor growth of imatinib-resistant KCL-22 clones in NSG mice. **(A)** A total of 1x10^6^ cells of individual resistant clones were subcutaneously inoculated into mice (n = 4 per clone). After three days, mice were randomly distributed into imatinib-treated (n = 2 per clone; 100 mg/kg in PBS 1 x per day by oral gavage; IM+; black columns) and untreated/control (n = 2 per clone; 200 µl PBS 1 x per day by oral gavage; IM-; white columns) groups and monitored for tumor detection. The X-axis represents days since transplantation. Bars represent detected and growing tumors. **(B)** Tumor growth of imatinib-resistant KCL-22R clones in NSG mice treated with imatinib. The X-axis represents days since the first tumor detection. The Y-axis represents tumor volume. Error bars represent standard deviations.

All 4 KCL-22R clones were able to engraft in the mice without any apparent difference between the imatinib-treated and untreated groups. Individual clones exhibited different engraftment abilities (**Figure 6A**). T315I-mutated Clone 1 exhibited the fastest growth in the imatinib-treated cohort, similar to *in vitro* conditions (**Figure 6B**). Similar data were observed in the imatinib-untreated cohort.

## Discussion

Both the selection of preexisting BCR-ABL1 mutated clones (15) and *de novo* acquisition during TKIs therapy (8, 9) were proposed to explain the emergence of TKI-resistant mutations. This study worked with the hypothesis of *de novo* acquisition of mutations conferring resistance to imatinib as the prevalent mechanism in CML cells.

The established *in vitro* model of CML KCL-22 cells has the ability to relapse after exposure to imatinib upon acquiring the T315I mutation (8). Despite differences in the procedure of establishing imatinib-resistant clones using single-cell FACS sorting, *de novo* acquisition of BCR-ABL1 KD and other somatic mutations was confirmed in this work by sensitive NGS and ASO-ddPCR. The same clonal population identically exposed to imatinib in parallel experiments was able to either develop different mutations or fail to acquire any mutation conferring resistance, thus proving the *de novo* acquisition process and supporting the hypothesis that the existence of the specific pool of pre-mutated or mutationally destined cells is unlikely for KCL-22 cells. *De novo* acquisition of BCR-ABL1 mutations was also confirmed in the CML-T1 model.

Recently, it was established that the genetic landscape of CML is significantly more diverse than assumed in the past, particularly in recurrent disease and advanced phases (6). Indeed, several mutated genes in addition to BCR-ABL1 were identified by the DNA NGS myeloid panel in resistant KCL-22R clones, namely, *BCOR*, *ATRX*, *RUNX1* and *KRAS*. All these genes were previously found to be mutated in CML, although their clinical importance for CML patients remains unclear (5, 16, 17). *RUNX1* was found to be commonly mutated during both CML-CP diagnosis and blast phase, often in cooccurrence with other recurrent mutations (17, 18). *RUNX1* mutations are associated with the block of cell differentiation and with aggressive AML (19, 20). Notably, KCL-22 carries isochromosome 21 [i(21)(q10)], possessing 3 alleles of *RUNX1*, and mutations in *RUNX1* were found to be associated with Chr21 trisomy (21). In the case of *KRAS*, *de novo* mutation acquisition was proven in the clone bearing mutations in *ATRX* and *RUNX1*. While *KRAS* mutations in CML seem to be particularly rare events (22), it should be noted that the pattern of acquisition of a *KRAS* mutation following *RUNX1* genetic lesions was described in AML (23). The association of the *BCOR* and *ATRX* mutations with prognosis remains contradictory in CML (5, 24). In contrast to KCL-22R clones, no somatic mutations beside BCR-ABL1 KD were identified in CML-T1R clones. However, it must be mentioned that some of the genes frequently found to be mutated in the lymphoid blast phase (e.g., IKZF1) (17, 25) were omitted in the NGS myeloid gene panel.

The exact molecular mechanism of mutagenesis of BCR-ABL1 KD and other oncogenes remains unclear. It was proposed that BCR-ABL1-induced reactive oxygen species (ROS) are responsible for genomic instability and BCR-ABL1 mutation acquisition in CML cells (26–28). While ROS may definitely increase the genomic instability of CML cells and contribute to deregulation of DNA repair (29), the proposed pathways of ROS induction *via* STAT5 and PI3K/Akt were found to be inhibited in KCL-22 and CML-T1 cells treated with imatinib and remained diminished even after mutation acquisition, making their contribution to *de novo* mutagenesis questionable.

Therefore, the DNA damage response and DNA repair genes with notably changed expression at the time of mutation acquisition were identified and subsequently annotated to the functions in the mismatch repair and BER pathways. Only a few studies are examining the role of mismatch repair in CML. Rather, an inhibitory effect of BCR-ABL1 on the expression of mismatch repair genes was found in CML cells (30). The data from expression arrays in this work revealed that the expression of most of the mismatch repair-specific genes was unchanged during mutagenesis. By contrast, the expression of genes involved in the long-patch variant of the BER pathway was significantly upregulated during mutation acquisition. FEN1 has been observed overexpressed in a broad spectrum of cancers and was previously associated with PCNA-dependent genetic instability and DNA damage (31, 32). The increased expression of POLD3 was also observed during mutation acquisition. The POLD3 subunit of DNA polymerase δ was found to induce mutagenesis by promoting translesion synthesis. POLD3 is required for inducing C-to-T mutagenesis in an abasic site (33). Notably, C-to-T base exchange is behind the T315I BCR-ABL1 mutation, which is frequently acquired in KCL-22R clones and KCL-22 cells and is one of the most common mutations in advanced CML.

In addition to their role in BER (34, 35), UNG and LIG1 are also associated with the error-prone alt-NHEJ repair pathway of DSB (36). Alt-NHEJ was originally depicted as a backup pathway in cells with defective c-NHEJ (37), while some recent studies suggest that inactivation of c-NHEJ is not required for alt-NHEJ activity (38). The group of Chen et al. (8, 9) proposed that KCL-22 cells utilize NAD+-dependent c-NHEJ to induce point mutations (14). Overexpression of CD38 NAD+ hydrolase leads to abrogation of BCR-ABL1 mutation acquisition (14). The data of this work do not provide strong support for c-NHEJ involvement in *de novo* mutation acquisition. First, in contrast to Wang et al., CD38+ resistant KCL-22R clones with BCR-ABL1 KD mutations were also isolated. This discrepancy may be caused by the different origins of CD38+ KCL-22 cells tested in both studies, which include naturally occurring CD38+ cell subpopulation vs. cells with artificial CD38 overexpression, which may lose or diminish the mutagenesis ability. Second, the expression of *LIG4*, *XRCC4* and *XRCC6* genes was not changed in the KCL-22 (and CML-T1) model, and DNA methylation associated with repressive chromatin marks was found in the regulatory regions of *LIG4*. However, it should be noted, that upregulation of *LIG4* expression, along with upregulation of alt-NHEJ genes, was observed during mutation acquisition in PB leukocytes of CML patients. Notwithstanding, data in general are in agreement with the studies proposing that the alt-NHEJ pathway is activated in TKI-resistant CML cells (10, 39). Increased expression of LIG1, UNG and PARP1 was found in cells undergoing mutagenesis. The expression of alt-NHEJ factors in CML was previously found to be augmented by the oncogenic transcription factor MYC (11). Accordingly, MYC is strongly expressed in both KCL-22R and CML-T1R clones and CML-resistant primary cells, as described previously (40), and its binding was enriched in the *LIG1* transcription start site (TSS) at the time of mutation acquisition.

The growth abilities of individual KCL-22R and CML-T1R clones were assessed in different environmental conditions *in vitro* and *in vivo* with the conclusion that clonal evolution is driven by both mutation-type specific oncogenic properties and additional genetic/cytogenetic lesions in concert with environmental selection pressures. At high imatinib concentrations, T315I-bearing Clone 1 showed superior growth over other KCL-22R clones, in agreement with the superior resistance associated with the T315I mutation (41). The growing abilities of individual KCL-22R clones only partially reflect kinase activity revealed by CyTOF, a fact already described by others (42, 43). Conversely, the finding that the activity of the main BCR-ABL1-associated signaling pathways in KCL-22R clones is generally lower than that in imatinib-naïve KCL-22 cells is in accordance with the fact that BCR-ABL1 mutations do not confer a growth advantage in the absence of imatinib (44).

Taken together, this study provides evidence for the responsibility of *de novo* mutation acquisition for acquired imatinib resistance in CML-CP. Imatinib has been the most used frontline TKI to combat CML-CP, and it likely will remain in this position in the future owing to the benefits of low toxicity and cost-effectiveness even after emerging therapy goal of treatment-free remission has been considered (45, 46). For CML-CP patients, no robust evidence has conclusively shown that BCR-ABL1 KD mutations conferring resistance to imatinib may already be detectable at diagnosis, which was also confirmed in this work (47, 48). Somatic mutations acquired *de novo* in the follow-up constitute a bulwark of lesions associated with refractory and progressed CML with poor outcomes (5).

The data support the considered role of the alt-NHEJ repair pathway (11) and provide the first hint that BER, particularly the long-patch variant, may be involved in the acquisition of somatic point mutations in oncogenes in CML. Contrary to some other natural *in vitro* models of BCR-ABL1 mutagenesis (49), BCR-ABL1 KD mutations acquired in resistant KCL-22R and CML-T1R clones belong among the mutations most commonly detected in both CML-CP and advanced CML (50). Moreover, the same mechanism of acquisition may be undergoing in the case of somatic mutations in other cancer genes, thus underlying the clinical relevance of the presented findings.

In conclusion, somatic mutations associated with resistance to imatinib are acquired in CML-CP *de novo*, thus early mutation detection is highly required to prevent progression because several therapeutic options exist for cases with mutated KD of BCR-ABL1. However, even when mutations in other cancer genes can be uncovered early by currently feasible NGS approaches, the resistance associated with other somatic mutations or in combination with mutated BCR-ABL1 remains therapeutically challenging, which will require highly individualized treatment management in the future.

## Data Availability Statement

The datasets presented in this study can be found in online repositories. The names of the repository/repositories and accession number(s) can be found below: BioProject PRJNA750307.

## Ethics Statement

The studies involving human participants were reviewed and approved by UHKT Ethics Committee. The patients/participants provided their written informed consent to participate in this study. The animal study was reviewed and approved by Animal Care and the Use Committee, First Faculty of Medicine, Charles University, Prague.

## Author Contributions

NC wrote the manuscript, performed experiments, evaluated data, and contributed to the interpretation of the results. VP performed the majority of the experiments, evaluated data, and contributed to the interpretation of the results. PB performed experiments, analyzed data, and revised the paper. JK performed NGS analysis and evaluated data. AL performed *in vivo* experiments. TK and DKuz designed and interpreted mass cytometry data. JB and SR performed cytogenetic analysis and interpreted data. DKar performed expression arrays. KR prepared samples for mass cytometry analysis and interpreted data. VK and MB performed cell tracking and cell cycle analysis. DKun evaluated clonal development. JL performed resistant clone establishment. HK and CS provided patient samples and clinical data. PK supervised the *in vivo* experiments. OH supervised mass cytometry and flow cytometry analysis, interpreted data and critically revised the manuscript. KMP designed experiments, coordinated the research, interpreted the results, and revised the paper. All authors contributed to the article and approved the submitted version.

## Funding

This study was supported by the Czech Science Foundation (GACR) project no. 18-18407 and the Czech Ministry of Health project no. 00023736 for the conceptual development of a research institute.

## Conflict of Interest

HK was a consultant for and received honoraria from Novartis. KMP was a consultant for and received honoraria from Incyte and Angelini.

The remaining authors declare that the research was conducted in the absence of any commercial or financial relationships that could be construed as a potential conflict of interest.

## Publisher’s Note

All claims expressed in this article are solely those of the authors and do not necessarily represent those of their affiliated organizations, or those of the publisher, the editors and the reviewers. Any product that may be evaluated in this article, or claim that may be made by its manufacturer, is not guaranteed or endorsed by the publisher.

## Abbreviations

Alt-NHEJ, alternative nonhomologous end-joining; BER, base-excision repair; BM, bone marrow; CML, chronic myeloid leukemia; c-NHEJ, classical nonhomologous end-joining; CP, chronic phase; DMR, deep molecular response; DSB, double strand break; ELN, European Leukemia Net; FC, fold change; IdU, 5-iodo-2′-deoxyuridine; KD, kinase domain; MMR, major molecular response; NGS, Next Generation Sequencing; PB, peripheral blood; TKIs, tyrosine kinase inhibitors; TSS, transcription start site; WT, wild type.
