# Supplementary material for: Somatic Mutations in Oncogenes Are in Chronic Myeloid Leukemia Acquired De Novo via Deregulated Base-Excision Repair and Alternative Non-Homologous End Joining
Source: Front Oncol. 2021 Sep 20;11:744373. doi: 10.3389/fonc.2021.744373 (PMC8488388; doi:10.3389/fonc.2021.744373)
Supplement: Supplementary file 1 [file DataSheet_1.pdf]

SUPPLEMENTARY METHODS

CML cell lines and cytogenetic analysis

The KCL-22 and CML-T1 cell lines were handled and cultivated in appropriate medium according to the recommendations of the supplier and were tested for Mycoplasma contamination using the MycoAlert PLUS detection kit (LT07-705; Lonza). Mycoplasma contamination was tested no later than one month after the cells were thawed. The number of passages distinguished between *de novo* (up to 30 passages postthawing) and progressed (imatinib-naïve long-term growing) KCL-22 cells reflected the progression of untreated CML. Cell lines and established resistant clones were characterized and verified by cytogenetic analysis using the mFISH 24 XCyte Kit (D-0125-120-DI; MetaSystems GmbH, Altlußheim, Germany) for karyotype determination and the XL BCR-ABL1 Dual-Fusion probe (D-5052-100-OG; MetaSystems GmbH) for detection of BCR-ABL1 genomic fusion. The KCL-22 cell line is characterized by two Philadelphia (Ph) chromosomes and by the karyotype:

51,X,del(X)(p21),+der(1)t(1;10),+6,+8,+8,t(9;22)(q34;q11),der(17)t(17;19),i(21)(q10),+der(22)(t(9;22)

The CML-T1 cell line is characterized by two derivative chromosomes 22 carrying BCR-ABL1 fusions and by the diploid karyotype:

46,XX,der(3)t(3;20),t(6;7)(q25;q36),del(11)(q23),del(20)(q12q13) with near tetraploid sideline.

No cell lines used in this work are listed in the database of commonly misidentified cell lines.

Isolation of imatinib-resistant CML clones

Imatinib-resistant KCL-22R clones were prepared by single-cell FACS sorting to 0.004 µM imatinib. The imatinib concentration was increased to 0.4 µM after 30 days and subsequently increased to 4 µM after the next 45 days of growing. Imatinib-resistant CML-T1R clones were prepared by single-cell FACS sorting to 0.004 µM imatinib. The imatinib concentration was increased to 0.04 µM after one month, followed by a subsequent gradual increase to 0.4 µM, 1 µM and finally up to 4 µM after 30-45 days of growing in each step. Samples were collected for RNA and DNA isolation during the development of resistant clones at specific time points.

Patients and samples

The presence of BCR-ABL1 KD mutations was retrospectively analyzed at non-optimal responding CML patients with BCR-ABL1 mutations at the time of resistance and with available total leukocyte RNA samples at the time of diagnosis (n=21) (Table 1a). The presence of mutations in other oncogenes was retrospectively analyzed at non-optimal responding CML patients with BCR-ABL1 mutations at the time of resistance and with available total leukocyte DNA samples at the time of diagnosis (n=24) (Table 1b). The bone marrow (BM) samples (n=11) of CML patients (n=8) were collected at the time of diagnosis (n=4), at the time of imatinib resistance (n=4; 2/4 paired) and at the time of response to imatinib (n=3; 1/3 paired). CD34+ cells were isolated from the BM samples by magnetic-activated cell sorting (MACS) using a CD34 MicroBead kit (130-097-047; Miltenyi Biotec, Bergisch Gladbach, Germany) and an autoMACS Pro Separator (Miltenyi Biotec). Additionally, samples of total leukocytes from the PB of CML patients (n=3) were collected at the time of diagnosis (n=3) and at the time of mutation development (n=2; paired with 2/3 samples from the time of diagnosis). Pooled samples of leukocytes from the PB were obtained from healthy donors (n=5). Total leukocyte samples were prepared by osmotic lysis of PB. Characterization of patients and samples is provided (Table 2).

In vivo modes

Tumor growth was recorded using a digital caliper, and mice were followed up to selected timepoints and euthanized. After euthanasia, BM cells were aspirated from the femur, and tumors were removed and mechanically homogenized to obtain individual cells for subsequent analysis.

BCR-ABL1 expression analysis

RNA quality and quantity were analyzed on a 2100 Bioanalyzer (Agilent Technologies, Santa Clara, CA, USA) and a NanoDrop ND-1000 spectrophotometer (Thermo Fisher Scientific). To measure the BCR-ABL1 and β-glucuronidase (GUSB) expression levels, 200 ng of RNA was transcribed using 200 Ul of M-MLV Reverse Transcriptase-l (#28025013; Thermo Fisher Scientific) and random hexamers. Reverse transcription (RT) reaction conditions were 37 °C for 60 min followed by 94 °C for 3 min. BCR-ABL1 transcript quantification was performed using standardized method (1) and recommended primers and probes.(2)

Droplet digital PCR

Mutations in BCR-ABL1 KD and *KRAS* were analyzed at the DNA level using ASO-ddPCR. DNA was isolated from leukemic cells using RLT Plus buffer and the AllPrep DNA/RNA Micro Kit (Qiagen) according to the manufacturer´s instructions. Reaction conditions were set according to the manufacturer´s instructions. Analysis was performed in technical duplicates. KCL-22-specific BCR-ABL1 genomic fusion was characterized by NGS as previously described (3) and quantified by ddPCR using primers specifically detecting genomic BCR-ABL1 of KCL-22 (BCR-ABL F KCL-22: 5´-GATGCTCTGTGCCTTGGAT-3´; BCR-ABL R KCL-22: 5´-ACAAGGTATGCAAATGGATGTAAC-3´; BCR-ABL Probe KCL-22 5´-CCCACTCCCGTCCTCATTACAGAA-3´). The DNA mutation level of BCR-ABL1 was calculated as mutation copy number (in 20 µl)/BCR-ABL1 fusion copy number (in 20 µl) * 100 (%). The mutation level of *KRAS* was calculated as mutation copy number (in 20 µl)/total *KRAS* copy number (in 20 µl) * 100 (%). The copy number for the albumin (ALB) gene was used to check the DNA load in the analyzed samples.

Gene expression PCR Arrays

RT of RNA was performed using an RT^2 First Strand Kit (ID: 330401; Qiagen) containing RE3 Reverse Transcriptase Mix. The preincubation with GE buffer eliminated the contaminating genomic DNA from the RNA samples. RT reaction conditions were 42 °C for 15 min followed by 95 °C for 5 min. RT-PCR conditions were set according to the manufacturer´s instructions: 95 °C for 10 min, (95 °C for 15 sec, 60 °C for 1 min) - 45x repeats. Arrays were performed using RT^2 qPCR SYBR Green Rox Mastermix (330522; Qiagen). The converted Ct values of specific (s) and control (c) amplified genes calculated using the $2^{-(Cts-Ctc)}$ equation were compared using Student's *t*-test. Ctc was determined as a mean Ct value for 4 control genes (B2M, GAPDH, RPLP0, HPRT1).

Chromatin immunoprecipitation

Formaldehyde cross-linked chromatin from $3x10^6$ KCL-22 and CML-T1 cells and imatinib-resistant KCL-22R and CML-T1R clones was prepared as described previously (4) using the following antibodies: Anti-MYC ([9E11] ab56; Abcam), Anti-histone H3K9Ac (07-352; Millipore - Merck KGaA, Darmstadt, Germany), Anti-Histone H3K36Me3 - ChIP Grade (ab9050; Abcam), Anti-histone H3K4Me1 - ChIP Grade (ab7766; Abcam), Anti-Histone H3K4Me3 – ChIP Grade (ab1012; Abcam) or control IgG (NI01; Calbiochem®, Merck). The enrichment quantification was performed as previously described.(5)

DNA methylation analysis

DNA methylation was measured using the MagMeDIP Kit (Cat. No. C02010020, Diagenode, Liège, Belgium). Methyl DNA IP assays were performed using DNA isolated from KCL-22 and CML-T1 cells or imatinib-resistant KCL-22R and CML-T1R clones, respectively. IP was performed with internal positive and negative controls (methylated and unmethylated DNA, respectively). The DNA was isolated using the DNeasy Blood and Tissue Kit (ID: 69504, Qiagen) and was immunoprecipitated according to the MagMeDIP protocol. qPCR was performed using the primer pairs covering the DNA loci of interest and the control primers provided in the kit.

Sanger sequencing

Amplicons of *KRAS* were prepared using forward (5´-AAAGGTACTGGTGGAGTATTTGA-3´) and reverse (5´-ACGAATATGATCCAACAATAGAGGT-3´) primers. Amplicons were purified using a QIAquick Purification Kit (ID: 28104, Qiagen). Sequencing was performed using BigDye™ Terminator v3.1 Cycle Sequencing Kit (cat. number 4337455; Thermo Fisher Scientific) following the manufacturer's manual. The products of sequencing were purified using DyeEx 2.0 Spin Kit (cat. number 63206, Qiagen), dried by SpeedVac Vacuum Concentrator SPD11V (Thermo Fisher Scientific) and diluted in 25 µl formamide. Sequencing analysis was performed using ABI PRISM 3500xL (Thermo Fisher Scientific). Sequence analysis and mutation identification were performed using Mutation Surveyor v5.0 software (SoftGenetics).

New Generation Sequencing (NGS)

The cDNA BCR-ABL1 KD was selectively amplified using forward (5´-GAAACTCCAGACTGTCCACA-3´; BCR exon 13) and reverse (5´-CCTTTTCCACTTCGTCTGAG-3´; ABL1 exon 9/10) primers. Amplicons were purified using a QIAquick PCR Purification Kit (ID: 28104, Qiagen) and quantified using a Quant-iT PicoGreen dsDNA Assay Kit (cat. Number P7589, Thermo Fisher Scientific) and diluted to 0.2 ng/µl. The libraries were prepared using the Nextera XT DNA Library Prep Kit (Illumina) and pooled in equimolar amounts, diluted to 2 nM, denatured with 0.1 N sodium hydroxide and loaded at 10 pM on a MiSeq sequencer using MiSeq Reagent Micro Kit V2 for 300 cycles (cat. number MS-102-2002, Illumina). The NextGENe software and the in-house bioinformatics tool NextDom were used for data processing, error filtering and mutation calling at significant levels (median 1.5% of a particular mutation in total BCR-ABL1 transcripts, range 1.0-3.7%; p-value ≤0.05).
The DNA custom-designed NGS panel library was prepared using the KAPA HyperCap Kit (cat. number KK8512; Roche) following the manufacturer's manual. The library concentration was determined using the KAPA Library Quantification Kit (cat. number KK4835; Roche). The 15 pM library was analyzed on a MiSeq sequencer using the MiSeq Reagent Kit V2 for 300 cycles. The limit of detection for calling mutations was 5% when the minimum coverage reached 500x per nucleotide.

Proliferation and cell cycle analyses

The determination of the relative proliferation rate of KCL-22 clones resistant to imatinib was performed by staining cells with CellTrace reagents (with the ability to covalently bind to intracellular amines) and subsequent analysis of cell mixtures by Sony SP6800 Spectral Analyzer (Sony Biotechnology) at selected time points. The individual clones were alternately stained by four individual CellTrace dyes and mixed to obtain 4 unique dye-clone combinations for each experimental condition. The CellTrace dyes used were ViaFluor SE Cell Prol. Kit(488) (cat. number Biot30086-T, Biotium, Inc., Fremont, CA, USA) (1:1666), CellTrace Far Red Cell Proliferation Kit, (cat. number C34572, Thermo Fisher Scientific) (1:1000), CellTrace Yellow Cell Proliferation Kit (cat. number C34573, Thermo Fisher Scientific) (1:500) and CellTrace Violet Cell Proliferation Kit (cat. number C34571, Thermo Fisher Scientific) (1:1666).

To assess the passage of cells through the cell cycle, the Maxpar Cell Cycle Panel Kit (cat. Number 201313, Fluidigm) for proliferation was used to determine the status of G0 (senescence), G1, S-phase, G2, and M-phase (mitosis). KCL-22R and CML-T1R-resistant clones were incubated with IdU for 30 min and washed. Cells were fixed at selected time points and cryopreserved for the following analysis by CyTOF. For the analysis of cell cycle phases, metal-conjugated antibodies provided by the manufacturer were used (CyclinB1_153Eu).

PARP1 inhibition with NU-1025

The role of PARP1 in *de novo* mutation acquisition was assessed *in vitro* at KCL-22 and CML-T1 cells using PARP1 inhibitor NU-1025 (N7287, Sigma-Aldrich - Merck). IC_{50} values of NU-1025 were determined for KCL-22 (400 µM) and CML-T1 (350 µM) cells. The cells were treated with NU-1025 in one-tenth of its respective IC_{50} concentration (e.g. 40 µM for KCL-22 and 35 µM for CML-T1).

<u>Preparation of samples for mass cytometry</u>

Cells (0.5-1x10^6) for CyTOF analysis were fixed for 15 min with FIX-I buffer (FLD-201065, Fluidigm), diluted with Maxpar Cell Staining Buffer (201068, Fluidigm) and preserved in 10% glycerol in fetal bovine serum. Collected cell samples were barcoded in Cell Staining Buffer using different CyTOF metal-conjugated antibodies against the HLA-I and CD45 antigens and combined into 1 tube per experimental condition/timepoint. The amount of moAbs labeled with metal isotopes needed for one test was estimated using titration. Cells were permeabilized by 80% ice-cold methanol for 30 minutes. The samples were centrifuged directly in methanol and washed twice with CSB. Antibody cocktail (Supplementary Table S3) was thawed on ice, centrifuged, the supernatant was added to the cell pellet and incubated for 30 min. The cell pellet was washed twice in CSB, fixed with 1ml of freshly diluted 1.6% formaldehyde (10 min) and centrifuged.

<u>Mass cytometry</u>

After incubation of cells with 125nM 191/193Ir in Maxpar® Fix and Perm Buffer (at least 12h, up to 2 weeks) cells were washed twice in 2ml of MP CSB and once with 1ml of Cell Acquisition Solution (CAS). The cell pellet was diluted up to 1x10^6/ml with 15% of EQTM Four Element Calibration Beads (Fluidigm) in CAS to and filtered through a 35 µm nylon mesh cell-strainer cap (BD Biosciences). The samples were acquired using Helios (Fluidigm) with CyTOF software, version 6.7.1014. The instrument was prepared for acquisition by tuning using Tuning Solution (Fluidigm) according to manufacturer's instruction. The noise reduction (the cell length 7–150, lower convolution threshold 200) was applied during the acquisition. The signal was normalized through a Fluidigm algorithm, which is based on the 'Bead Passport' concept. The expression of proteins in non-apoptotic cells - PARP(cleaved) negative, Caspase3 (cleaved) negative – was measured.

<u>SEC-MAP analyses of protein expression</u>

Size exclusion chromatography-microsphere-based affinity proteomics arrays (SEC-MAP) containing 576 different antibodies against cellular proteins was used to analyze protein expression per sample of KCL-22 progressed cells as previously described.(6)

<u>Statistical analysis</u>

K-mean clustering of gene expression profiles was performed based on Pearson correlation distance. MeV4 software (Multiple Experiment Viewer; http://mev.tm4.org/) (7) was used to visualize the gene expression profiles as heatmaps.

SUPPLEMENTARY RESULTS

De novo acquisition of mutations was confirmed in CML-T1R clones

To confirm the observation of KCL-22 cells with the ability to acquire imatinib-resistant mutations *in vitro*, imatinib-resistant CML-T1R clones were isolated by single-cell FACS of imatinib-naïve CML-T1 cells with a subsequent increase in imatinib concentration up to 4 µM. Three CML-T1R clones resistant to 4 µM imatinib were established. Two out of three CML-T1R clones originated from CD38+ cells and 1 of 3 originated from CD38- cells (Supplementary Figure S1b). Mutational analysis uncovered in Clone 1: Y253H (50%); Clone 2: G250E (50%) + Y253H (31%) + F359C (23%) and Clone 3: Q252H (50%) + E459K (45%) BCR-ABL1 transcripts. BCR-ABL1 mutations started to be detected beginning on day 105 post-sorting at the 0.4 µM level (Supplementary Figure S3). In Clone 1, the competition of subclones with an acquired BCR-ABL1 mutation was observed during culturing at 0.4 µM imatinib with subsequent expansion of Y253H-positive clone over clones with M351T and E450G mutations following the imatinib dose escalation (Supplementary Figure S3). No other mutated genes were detected by NGS myeloid panel analysis. No additional cytogenetic aberration or additional BCR-ABL1 amplification was present in CML-T1R clones.

The expression profiles of KCL-22R at the time of mutation acquisition were different from those of the KCL-22S clones

The expression profiles were studied in 4 KCL-22R clones and 4 KCL-22S clones (imatinib-naïve; controls) on days 60-75 post-sorting, when BCR-ABL1 and KRAS mutations in resistant clones were first detected by NGS or ASO-ddPCR. Imatinib-resistant and imatinib-sensitive clones clustered separately according to the gene expression profiles at this time point (Supplementary Figure S4a). However, separate clustering between resistant and sensitive clones was not observed on day 90 after sorting, suggesting that the DNA damage response and/or DNA repair mechanisms were no longer active since the mutations were established (data not shown).

Increased transcription of BER and alt-NHEJ genes at the time of mutation acquisition was confirmed in the KCL-22R´ and CML-T1 clones

Increased expression of BER and alt-NHEJ genes was also confirmed at the time of mutation acquisition in the imatinib-resistant KCL-22R´ clones (Clone 2´; Clone 3´), which were newly isolated from the preserved sorted cells, in comparison to imatinib-exposed clones (Clone 2´´´; Clone 3´´), which failed to redevelop resistance (Supplementary Figure S5b). To validate the suggested role of BER (long patch) and alt-NHEJ pathways in mutagenesis, the expression of *UNG*, *FEN1*, *POLD3*, *PCNA*, *LIG1* and *PARP1* was also determined in CML-T1R-resistant clones at the time the first mutation was detected by NGS on days 105/150 after sorting and was compared with their sensitive counterparts. Except that of *PARP1*, the expression of these genes was confirmed to be increased (Supplementary Figure S5c).

alt-NHEJ genes are epigenetically activated at the KCL-22R and CML-T1R resistant clones and mutation acquisition is accompanied by increased MYC occupation in regulatory region of *LIG1*

To put transcriptional data into a broader cell-regulatory context, the regions of DNA methylation and histone modifications of the *PARP1, LIG1* and *LIG4* genes were predicted using ChIP-Seq data from the UCSC Genome Browser (http://genome.ucsc.edu/index.html) (Supplementary Figure S6). Investigating chromatin structure in the transcription start sites (TSS) of the *PARP1, LIG1* and *LIG4* genes in KCL-22R clones, significant enrichment of the H3K4Me1 and H3K36Me3 histone marks, which are associated with transcriptionally active chromatin, was found in the TSSs of *LIG1* and *PARP1* but not of *LIG4* in the KCL-22R clones compared to that in the KCL-22S clones (Supplementary Figure S7a-b). No significant differences were found for the H3K9Ac and H3K4Me3 histone marks also associated with

transcriptionally active chromatin (Supplementary Figure S7c-d). The DNA methylation statuses of the TSSs were determined and compared between the KCL-22R clones and imatinib-naïve KCL-22 cells. Unmethylated DNA was found in *PARP1* and *LIG1*, while CpG of the TSS of the *LIG4* gene was significantly more methylated in resistant clones than in sensitive KCL-22 cells, consistent with data from transcriptional and ChIP analysis, suggesting inhibition of LIG4 expression during resistance development (Supplementary Figure S7e). Next, chromatin structure in the TSS of the *PARP1, LIG1* and *LIG4* genes was explored in KCL-22 cells exposed to 0.4 µM imatinib for 5 weeks undergoing mutation acquisition (BCR-ABL1 Y253H) in comparison to that in unmutated *de novo* KCL-22 cells. The presence of H3K4Me1 histone mark was decreased in the TSS of *LIG4* (Supplementary Figure S7f), while the H3K36Me3 was not significantly changed (Supplementary Figure S7g). Moreover, MYC binding was found to be significantly enriched in the TSS of *LIG1,* but not in that of *LIG4* or *PARP1*, in KCL-22 cells undergoing mutagenesis (Supplementary Figure S7h). H3K4Me1 and H3K36Me3 enrichment covering the TSS of *LIG1* and *PARP1* was also observed in CML-T1R-resistant clones (Supplementary Figure S8a-b).

<u>PARP1 inhibitor NU-1025 prevented or delayed BCR-ABL1 mutation acquisition in leukemic cells after imatinib treatment *in vitro*</u>

KCL-22 and CML-T1 cells were exposed to growing concentrations of imatinib for 7 weeks and the presence of BCR-ABL1 mutations was determined by NGS in a) cells co-treated with PARP1 inhibitor NU-1025 and b) NU-1025-untreated cells. In CML-T1 cells, NU-1025 treatment prevented BCR-ABL1 mutation acquisition and the unmutated and non-resistant cells died after the exposure to 1 µM imatinib in week 6 of the culture. In KCL-22 cells, BCR-ABL1 T315I mutation acquisition was not completely prevented, however the first detection of mutation by NGS was delayed in NU-1025 treated cells (Supplementary figure S9).

<u>After *de novo* mutation acquisition, the clonal evolution of resistant CML cells is dependent on the time and dose of imatinib</u>

To study the process of BCR-ABL1 KD mutation development in models of natural CML evolution, *de novo* KCL-22 cells were exposed to imatinib in six independent experiments (i) – (vi). Invariably, no BCR-ABL1 KD mutations were detected in imatinib-naïve KCL-22 cells. However, BCR-ABL1 mutations were repeatedly detected by NGS after exposure of *de novo* cells to 0.4 µM imatinib for 30 days. T315I was detected in 5/6 experiments, and E255K, Y253H and H396R were detected in 1/6 experiment each (Supplementary Figure S10a). Mutations were detected as early as 14 days after exposure to imatinib in 2/6 experiments. Similarly, six independent experiments of CML-T1 cells exposed to 0.4 µM imatinib were performed. No BCR-ABL1 mutations were detected by NGS in imatinib-naïve cells. In 3/6 experiments, E255V mutations, and in 3/6 experiments, G250E mutations were detected. To study the expansion of BCR-ABL1 mutant KCL-22 cells in dependency on imatinib concentration and the time of exposure in detail, experiment (i) was processed with the subsequently increasing imatinib concentrations up to 4 µM and with a total follow-up of 350 days. During the first culture period (up to 90 days already in 4 µM imatinib), T315I-positive cells expanded, whereas E255K transcripts became undetectable. However, since day 90 of 4 µM imatinib, E255K transcripts reemerged and increased over time to 100%, while the T315I transcript decreased to undetectable levels (Supplementary Figure S10b). The E255K mutation detected at 100% on both the mRNA and DNA levels indicates the acquisition of this type of mutation on both Ph chromosomes of KCL-22 cells. BCR-ABL1 expression was determined during the follow-up period, showing overexpression of BCR-ABL1 in KCL-22 cells in general with a further sharp increase in BCR-ABL1 levels during the imatinib dose escalation followed by its gradual decline (Supplementary Figure S10b). The KCL-22 subclone with T315I was outgrown in the culture by the subclone with E255K in reaction to the imatinib concentration decreasing faster (Supplementary Figure S10c) than the concentration was increasing (Supplementary Figure S10b).

The delay of mutation acquisition and activation of BCR-ABL1-independent mechanisms was found in KCL-22 progressed cells

Unlike *de novo* KCL-22 cells (defined as growing up to 30 passages post-thawing), progressed KCL-22 cells (defined as imatinib-naïve long-term growing cells) developed resistance after exposure to 0.4 µM imatinib without acquiring BCR-ABL1 KD mutations in a follow-up period of 60 days. Progressed KCL-22 showed a higher IC50 value for imatinib (0.635 µM) than *de novo* KCL-22 (0.382 µM). Neither gene amplification nor BCR-ABL1 overexpression (Supplementary Figure S11a-b) was presented in these cells to explain the observed difference in imatinib sensitivity. Using the SEC-MAP protein array, considerably upregulated HIF1α and IGF1 protein expression and downregulated BAD and BID levels were identified 30 days after exposure to imatinib (Supplementary Figure S11c). Since increased protein expression of HIF1α and IGF1R is known to ensure proliferation, while decreased expression of the proapoptotic proteins BAD and BID enhances the survival of CML cells in the presence of TKIs, these data suggest that progressed KCL-22 cells are imatinib-resistant independent of BCR-ABL1.

Diminished activation of the BCR-ABL1-dependent pathways and persistent expression of MYC/BCL-2 were found in imatinib-resistant clones

The signaling properties of mutated clones responsible for their resistance were studied using a CyTOF custom-designed panel (Supplementary Table S3). Analysis of the protein levels was performed on the parental KCL-22 and CML-T1 cells treated by imatinib (Supplementary Figure S14), KCL-22 clones growing in 0.004 µM imatinib 21 days after sorting (Supplementary Figure S15), the resistant KCL-22R clones (Supplementary Figure S16) and resistant CML-T1R clones (Supplementary Figure S17) either growing in 4 µM imatinib or 1 week after imatinib withdraw. Similar protein expression's profiles were found between parental cells treated by imatinib and also between KCL-22 clones 21 days after sorting. The analysis showed inhibition of the major signaling pathways of BCR-ABL1 upon imatinib treatment, while the expression of MYC and BCL-2 was rather maintained (Supplementary Figures S14-S15). KCL-22 clones growing in 0.004 µM imatinib 21 days post-sorting (no mutation was detected at this time point) displayed stable or elevated expression of activated pMAPK11 (p38) kinase and its downstream target pCREB (Supplementary Figure S15), in concordance with previously published data.(8,9) BCR-ABL1-mutated KCL-22R-resistant clones growing in 4 µM imatinib displayed only partial activation of the STAT5, Erk1/Erk2 and AKT signaling in comparison to imatinib-naïve KCL-22 cells. Preferential activation of STAT5 signaling and downregulation of the ERK1/ERK2 and pAKT pathways were observed after imatinib withdrawal (Supplementary Figure S16). KCL-22R Clone 3 with the KRAS mutation revealed a relatively low level of pSTAT5 and BCL-2 and increased expression of the pERK1/ERK2 and pAKT proteins (downstream effectors of KRAS signaling) compared to clones with BCR-ABL1 mutations irrespective of cultivation condition (Supplementary Figure S16). In addition to persisting high levels of MYC, CML-T1R-resistant clones strongly overexpress the BCL-2 protein (Supplementary Figure S17).

SUPPLEMENTARY REFERENCES

1. Müller MC, Cross NCP, Erben P, Schenk T, Hanfstein B, Ernst T, et al. Harmonization of molecular monitoring of CML therapy in Europe. Leukemia. 2009;23(11):1957-1963.
2. Gabert J, Beillard E, van der Velden, VHJ, Bi W, Grimwade D, Pallisgaard N, et al. Standardization and quality control studies of 'real-time' quantitative reverse transcriptase polymerase chain reaction of fusion gene transcripts for residual disease detection in leukemia - a Europe Against Cancer program. Leukemia. 2003;17(12):2318-2357.
3. Machova Polakova K, Zizkova H, Zuna J, Motlova E, Hovorkova L, Gottschalk A, et al. Analysis of chronic myeloid leukaemia during deep molecular response by genomic PCR: a traffic light stratification model with impact on treatment-free remission. Leukemia. 2020;34(8):2113-2124.

4. Burda P, Curik N, Kokavec J, Basova P, Mikulenkova D, Skoultchi AI, et al. PU.1 activation relieves GATA-1-mediated repression of Cebpa and Cbfb during leukemia differentiation. Mol Cancer Res. 2009;7(10):1693-1703.
5. Papadopoulos GL, Karkoulia E, Tsamardinos I, Porcher C, Ragoussis J, Bungert J, et al. GATA-1 genome-wide occupancy associates with distinct epigenetic profiles in mouse fetal liver erythropoiesis. Nucleic Acids Res. 2013;41(9):4938-4948.
6. Kanderova V, Kuzilkova D, Stuchly J, Vlaskova M, Brdicka T, Fiser K, et al. High-resolution Antibody Array Analysis of Childhood Acute Leukemia Cells. Mol Cell Proteomics. 2016;15(4):1246-1261.
7. Saeed AI, Sharov V, White J, Li J, Liang W, Bhagabati N, et al. TM4: a free, open-source system for microarray data management and analysis. Biotechniques. 2003;34(2):374-378.
8. Yu C, Krystal G, Varticovksi L, McKinstry R, Rahmani M, Dent P, et al. Pharmacologic mitogen-activated protein/extracellular signal-regulated kinase kinase/mitogen-activated protein kinase inhibitors interact synergistically with STI571 to induce apoptosis in Bcr/Abl-expressing human leukemia cells. Cancer Res. 2002;62(1):188-199.
9. Dumka D, Puri P, Carayol N, Lumby C, Balachandran H, Schuster K, et al. Activation of the p38 Map kinase pathway is essential for the antileukemic effects of dasatinib. Leuk Lymphoma. 2009;50(12):2017-2029.

Supplementary Figure S1

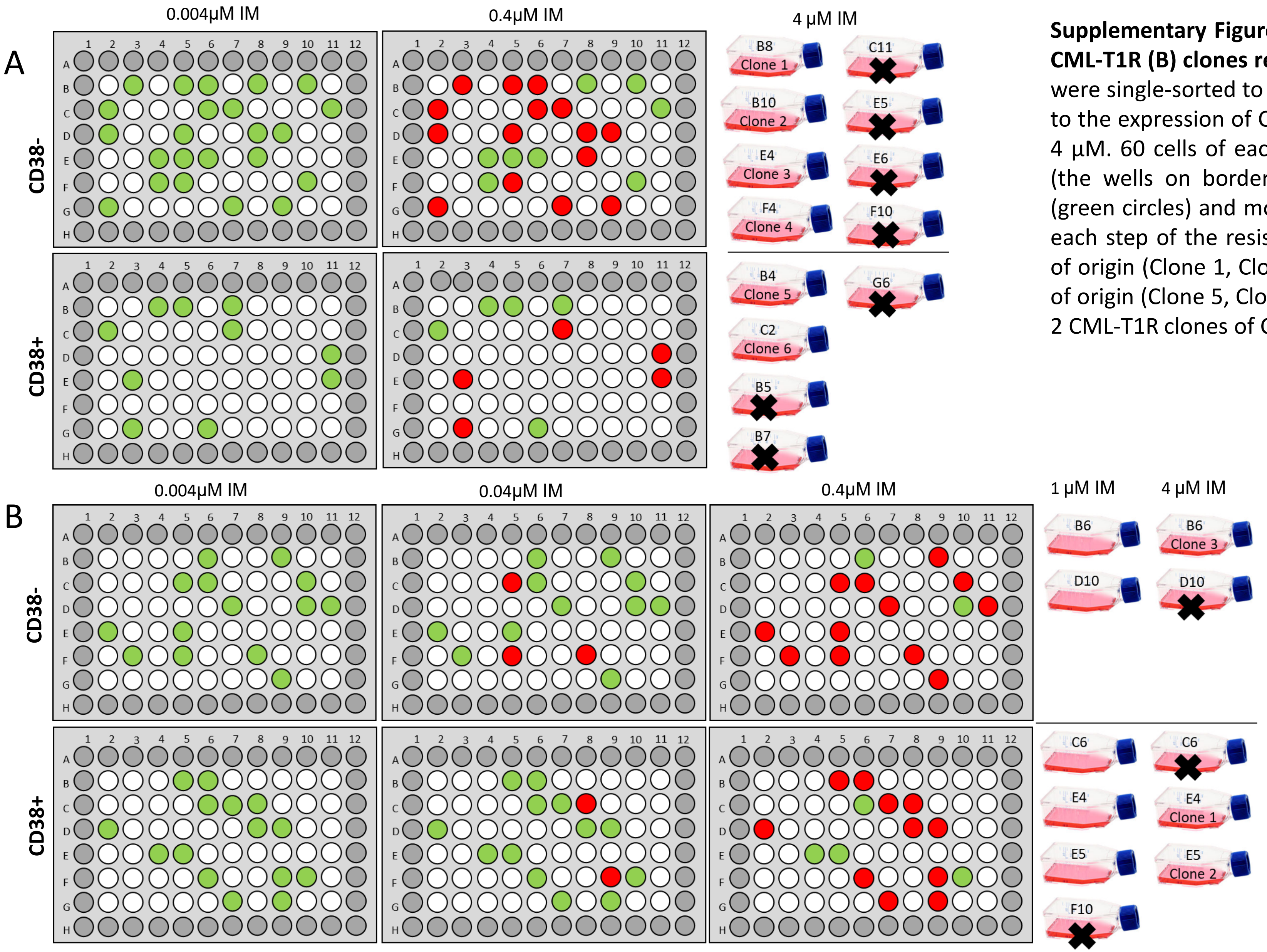

Supplementary Figure S1. **The scheme of the development of KCL-22R (A) and CML-T1R (B) clones resistant to 4 µM imatinib**. The KCL-22 and CML-T1 cell lines were single-sorted to 96-well plates with low concentration of imatinib according to the expression of CD38. Imatinib concentration was gradually increased up to 4 µM. 60 cells of each CD38- and CD38+ subsets for each cell line were sorted (the wells on borders of the plate were omitted – gray circles) Proliferating (green circles) and moribund (red circles; black X marks) clones are indicated for each step of the resistance establishment. Final, 4 KCL-22R clones of CD38- cell of origin (Clone 1, Clone 2, Clone 3 and Clone 4), 2 KCL-22R clones of CD38+ cell of origin (Clone 5, Clone 6), 1 CML-T1R clone of CD38- cell of origin (Clone 3) and 2 CML-T1R clones of CD38+ cell of origin (Clone 1, Clone 2) were established.

Supplementary Figure S2

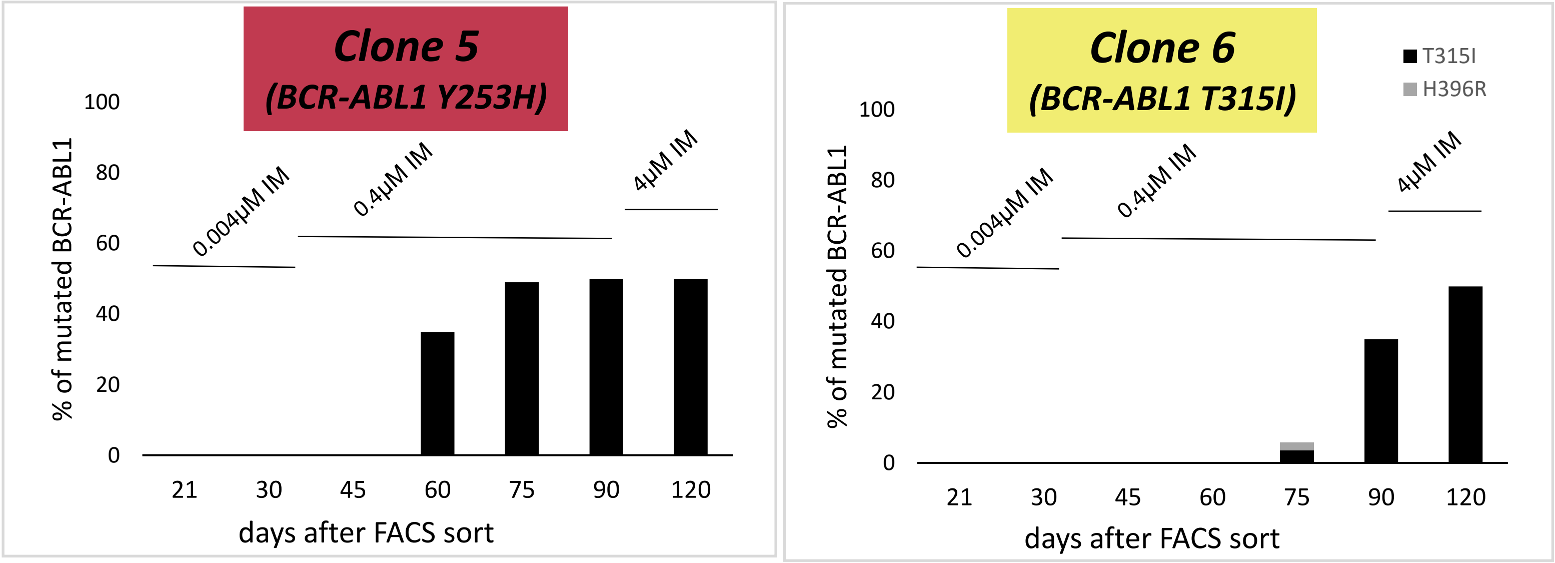

Supplementary Figure S2. ***De novo* acquisition of BCR-ABL1 KD mutations during the development of KCL-22 clones of CD38+ cell of origin resistant to 4 µM imatinib.** KCL-22R clones were prepared by single cell FACS sorting into medium with 0.004 µM imatinib. The imatinib concentration was increased to 0.4 µM in day 31 and further increased to 4 µM in day 75 after sort. The presence of mutated mRNA during the development of resistant KCL-22R clones up to day 120 post single-cell FACS sorting is shown: Clone 5 and Clone 6. The growing concentration of imatinib is indicated above the columns.

Supplementary Figure S3

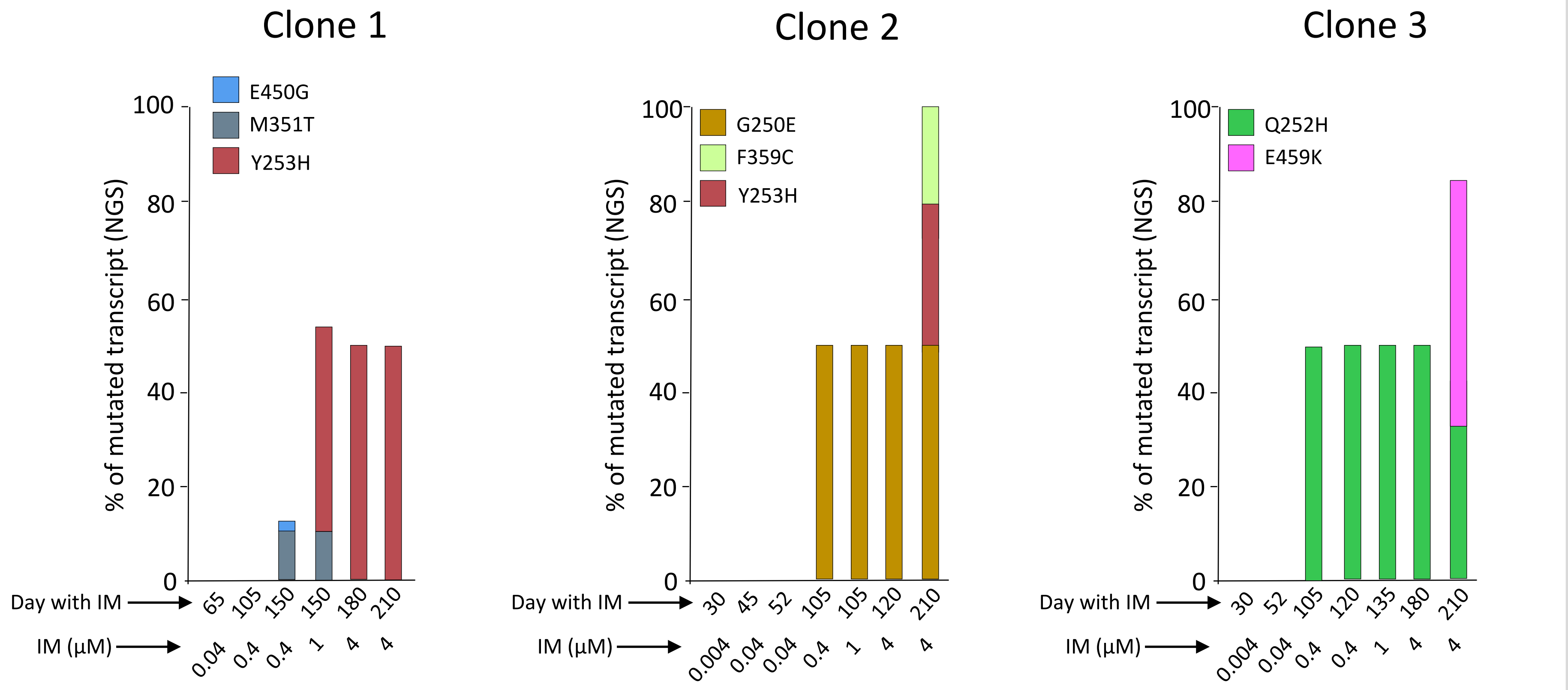

Supplementary Figure S3. ***De novo* acquisition of somatic mutations during the development of CML-T1R clones resistant to 4 µM imatinib**. The presence of mutated BCR-ABL1 mRNA (in % of total BCR-ABL1 transcript) during the development of resistant CML-T1R clones up to day 210 post single-cell FACS sorting: Clone 1, Clone 2 and Clone 3. The growing concentrations of imatinib are indicated below the X-axis. IM= imatinib.

Supplementary Figure S4

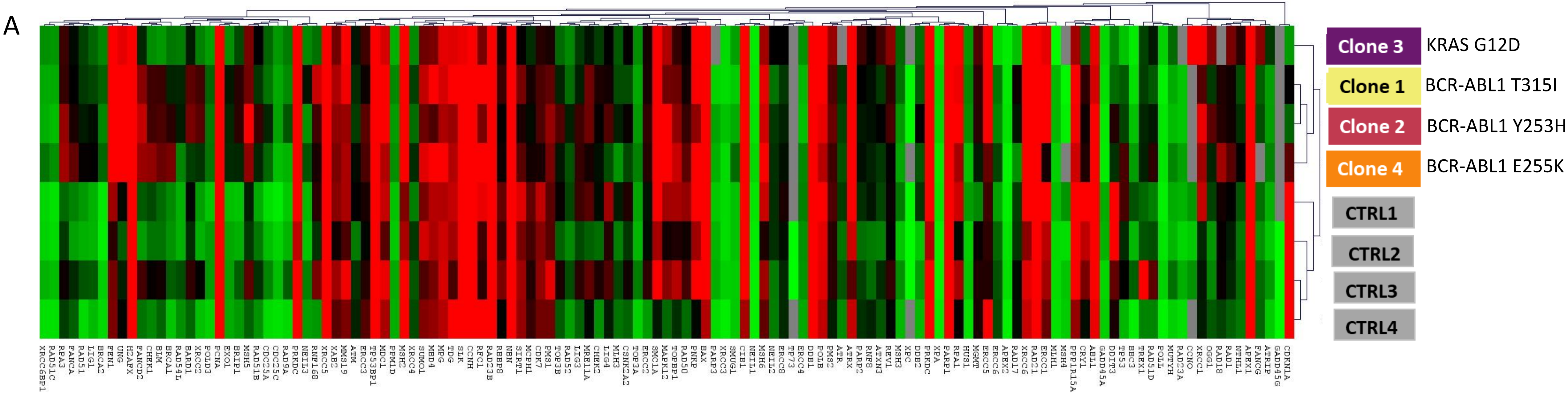

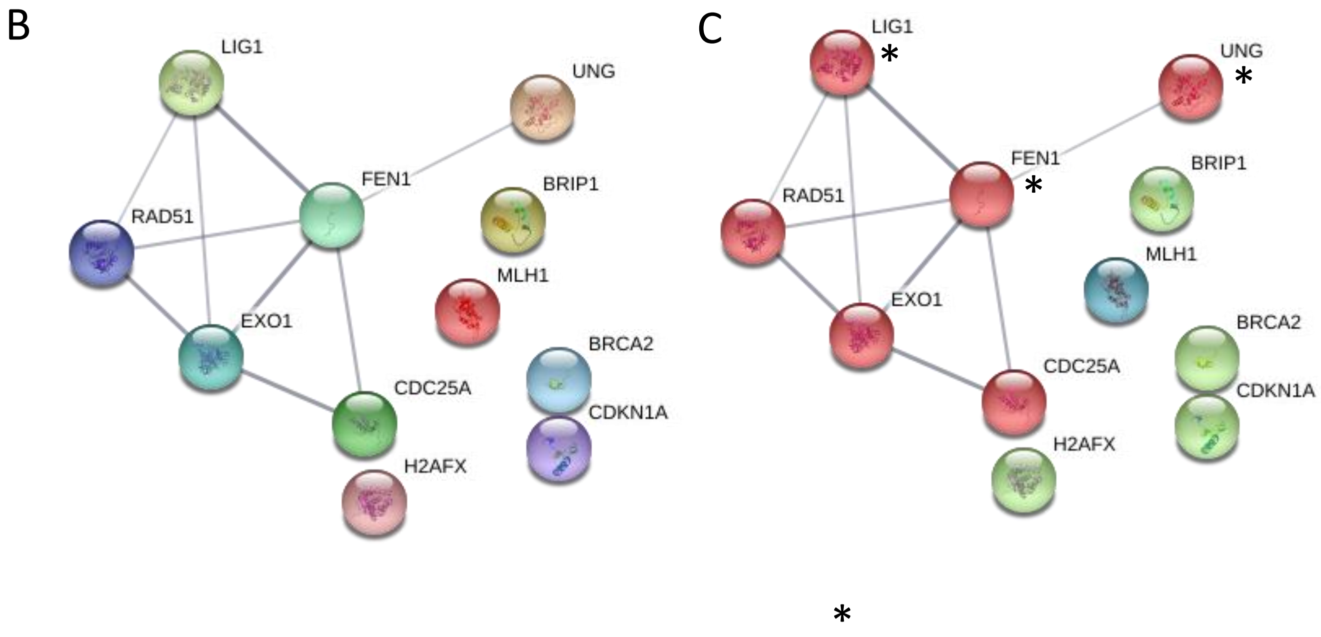

Supplementary Figure S4. The expression profiles of DNA damage response and DNA repair genes in imatinib-resistant KCL-22R (Clone 1 – Clone 4) and imatinib-sensitive (CTRL1 – CTRL4) KCL-22 clones. (A) The gene expression profiles of KCL-22R and KCL-22S clones were determined on days 60-75 after the cell sort representing the time of first mutation detection in KCL-22R clones by NGS. The gene expression levels of the studied molecules representing $2^{-(Cts-Ctc)}$ values are marked on a green-red color scale, covering 80% quantile of expression values. The gene clusters and sample clusters were calculated based on the Pearson correlation coefficient. (**B**) The connectivity network of proteins based on coexpression and cooccurrence parameters in STRING 11.0 (https://string-db.org/). The illustrated connecting edges represent "confidence" (strength of data support). Inclusion criteria for differentially expressed genes: 1) $2^{-\Delta Ct}$ mean value for KCL-22R resistant clones/$2^{-\Delta Ct}$ mean value for KCL-22S control clones should be ≤ 0.4 or ≥ 2.5 and 2) the difference in expression between the group of KCL-22R resistant clones and control clones should be statistically significant ($p < 0.05$) **(C)** K-means clustering of proteins coded by differentially expressed genes, indicated in STRING 11.0 as red, green and blue clusters. Genes involved in BER pathway are marked by star mark (*)

Supplementary Figure S5

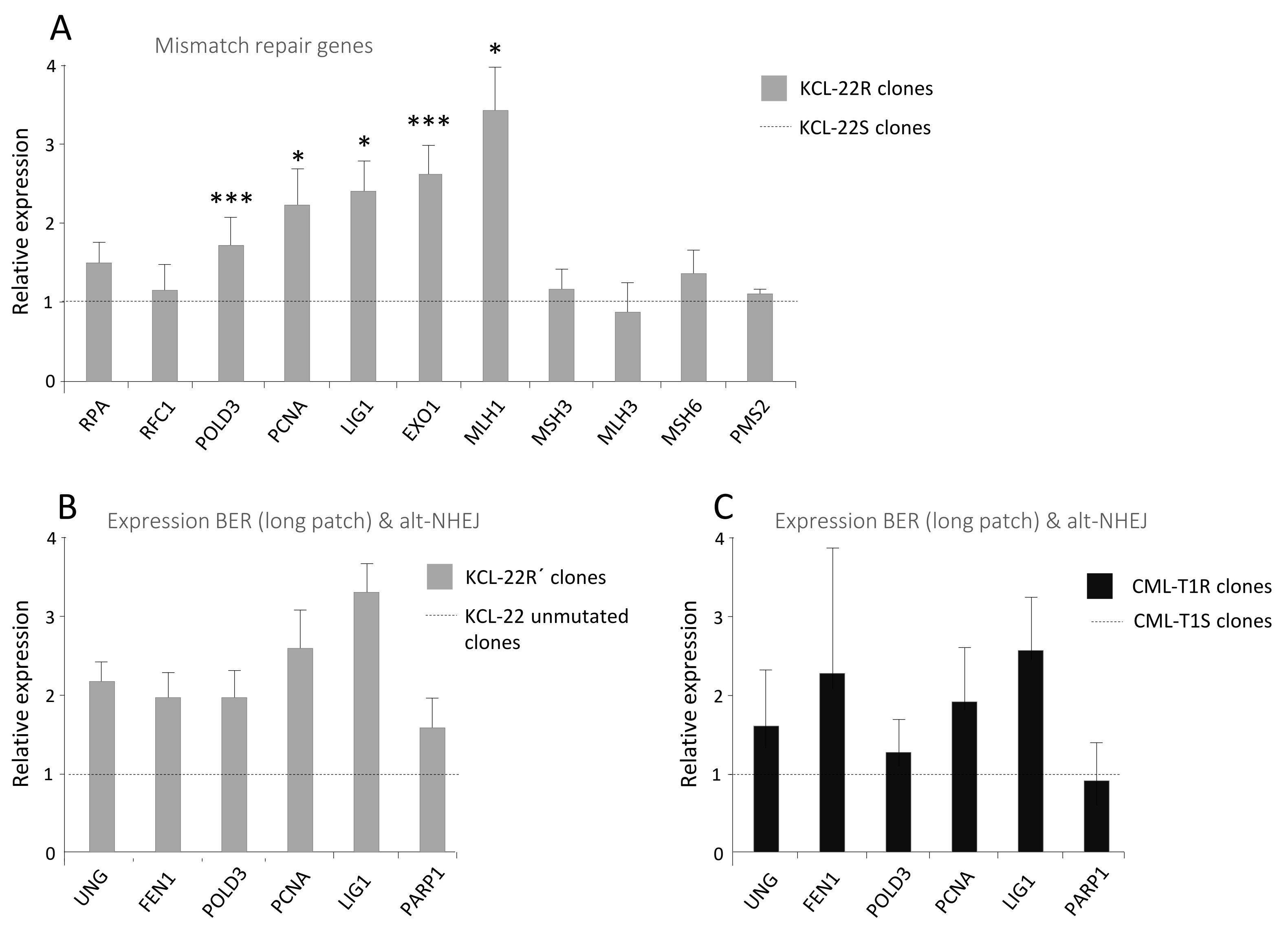

Supplementary Figure S5. The expression of DNA damage response and DNA repair genes in KCL-22R, KCL-22R´ and CML-T1R clones. The expression data are shown for the genes of mismatch repair **(A)** long-patch BER and alt-NHEJ **(B, C)** pathway at the days of mutation acquisition. Gray columns represent **(A)** the fold change (FC; relative expression) of average gene expression for KCL-22R-resistant clones in comparison to imatinib-sensitive KCL-22S clones or **(B)** the FC (relative expression) of average gene expression for KCL-22R´ clones (Clone 2´; Clone 3´), newly isolated from the preserved sorted cells, in comparison to imatinib-exposed but unmutated clones (Clone 2´´´; Clone 3´´). **(C)** Black columns represent the FC (relative expression) of average gene expression for CML-T1R-resistant clones compared to expression in control, imatinib-naïve CML-T1S clones. Expression in control clones is equalized to 1 and marked by the dashed line. The level of significance is indicated: * P<0.05 and *** P<0.001). Error bars represent standard deviations.

Supplementary Figure S6

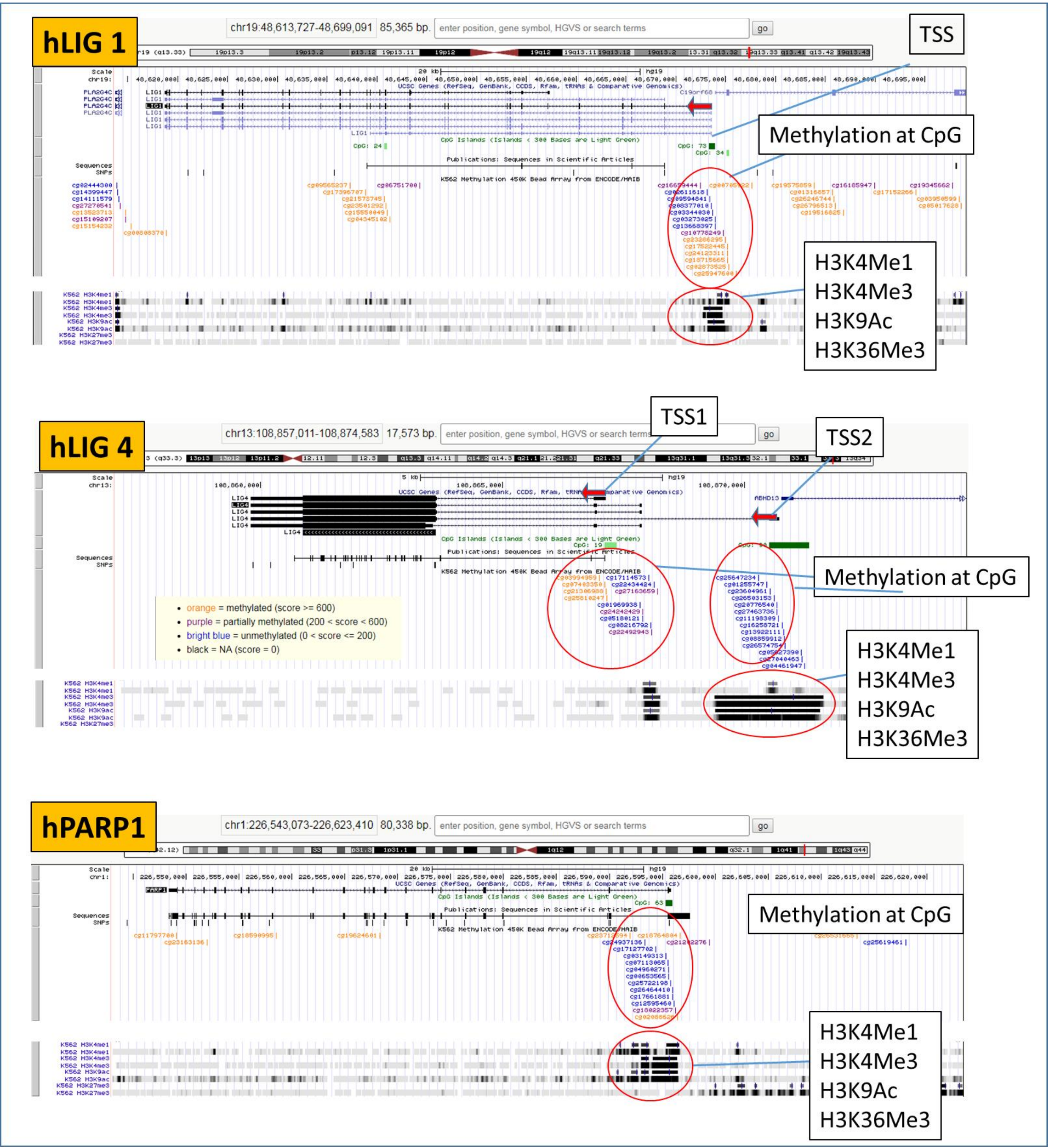

Supplementary Figure S6. The regions of putative epigenetic events in *LIG1, LIG4* and *PARP1* genes (ChIP-Seq from UCSC). Filled red circles mark regulatory loci putatively playing roles in the DNA methylation and histone modifications associated with active chromatin. The red arrows indicate the TSSs of the examined genes.

Supplementary Figure S7

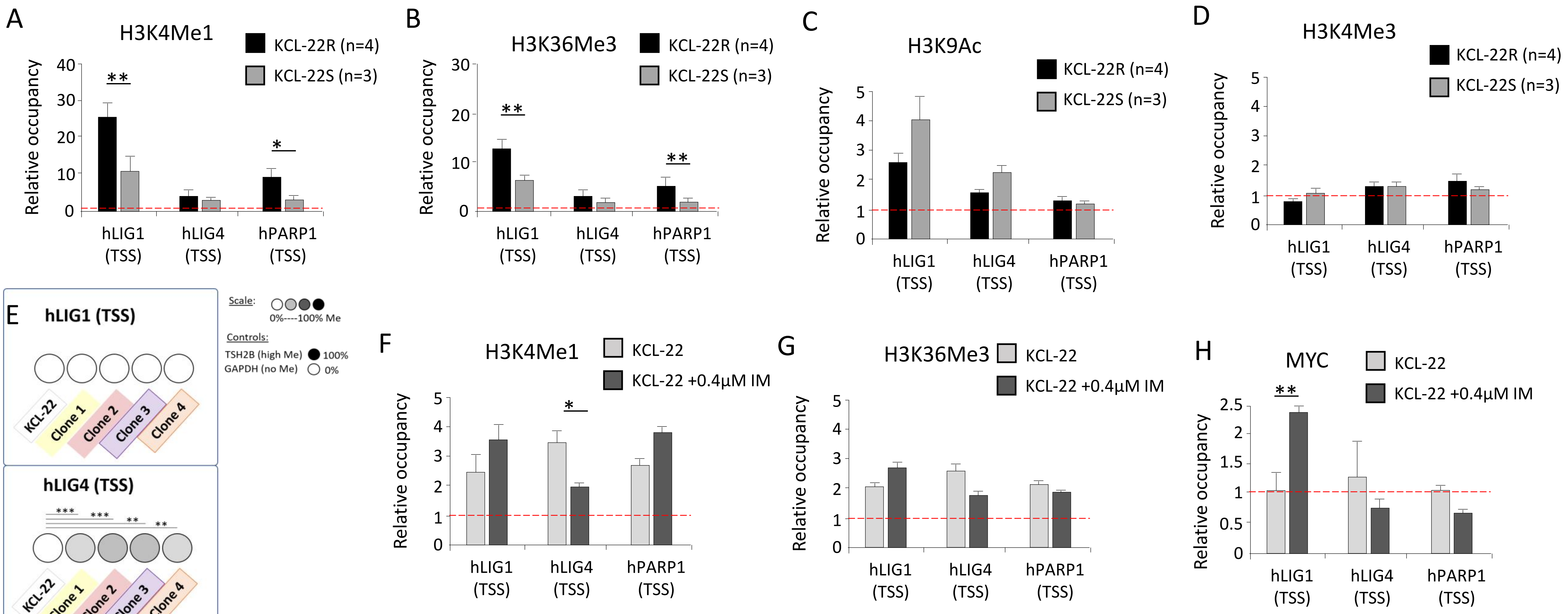

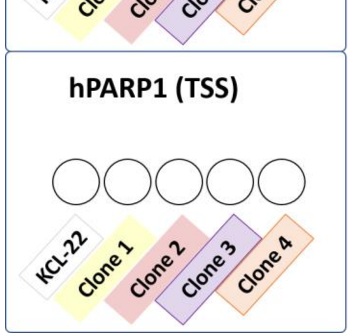

Supplementary Figure S7. Epigenetic events and MYC binding to the TSS of the *LIG1, LIG4* and *PARP1* genes in KCL-22 cells and KCL-22R clones. Dark gray columns represent the relative average occupancy of **(A)** H3K4Me1**, (B)** H3K36Me3, **(C)** H3K9Ac and **(D)** H3K4Me3 histone marks in KCL-22R clones in comparison to that in KCL-22S clones represented by light columns. **(E)** DNA methylation at the CpGs in the TSSs of *LIG1*, *LIG4* and *PARP1* was determined for KCL-22R clones in comparison to imatinib-sensitive naïve KCL-22 cells. DNA methylation is indicated as the percentage of CpG methylation relative to the positive control (PC) locus. PC - TSH2B: testes-specific H2B histone gene (hypermethylated in all tissues excluding testes); Negative control: GAPDH promoter. Average **(F)** H3K4Me1 and **(G)** H3K36Me3 histone marks occupancy and **(H)** MYC binding in naïve KCL-22 cells (light columns) in comparison to imatinib-treated KCL-22 cells at the time of BCR-ABL1 mutation (Y253H) acquisition (dark columns). * P<0.05, ** P<0.01 and *** P<0.001. Unspecific occupancy by IgG is equal to 1 and marked by the red dashed line. Error bars represent standard deviations.

Supplementary Figure S8

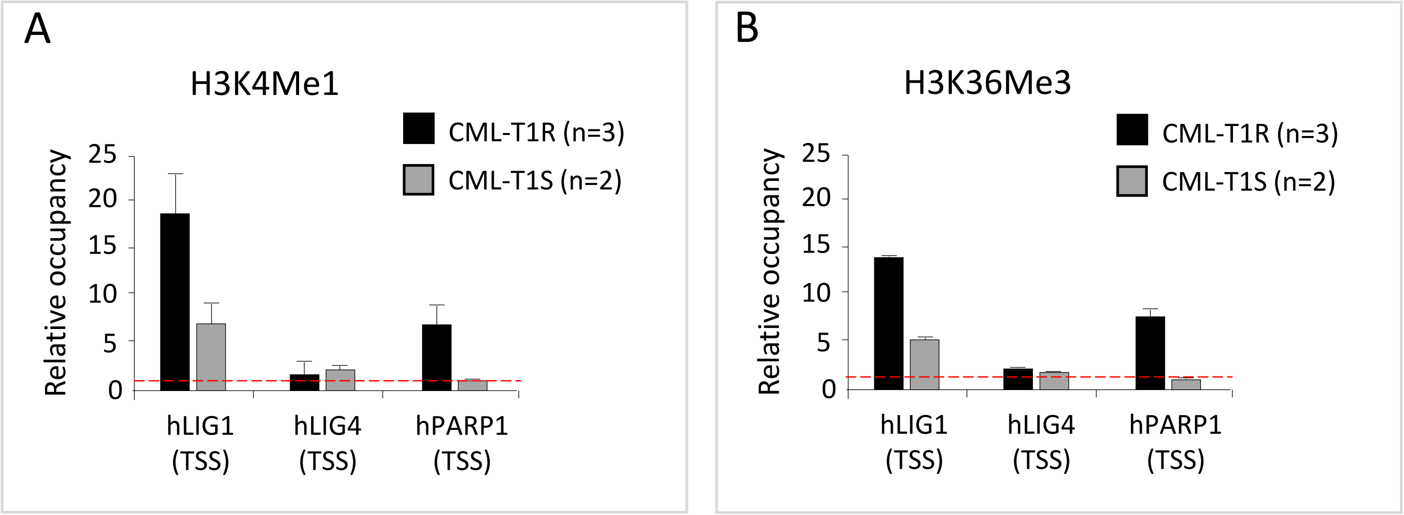

Supplementary Figure S8. Epigenetic events in the regulatory regions of the *LIG1, LIG4* and *PARP1* genes during mutation acquisition in CML-T1R clones. Black columns represent the relative average occupancy of **(A)** H3K4Me1 and **(B)** H3K36Me3 histone marks in CML-T1R clones in comparison to that in CML-T1S clones represented by gray columns. Unspecific occupancy by IgG is equal to 1 and marked by the red dashed line. Error bars represent standard deviations.

Supplementary figure S9

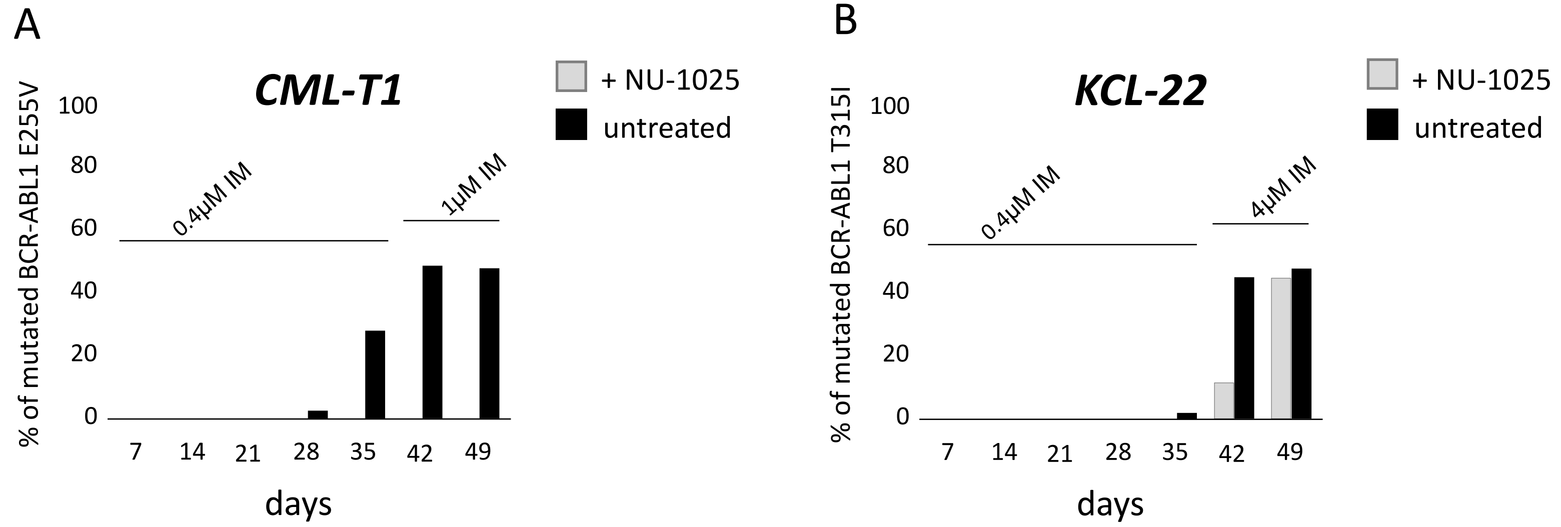

Supplementary figure S9. ***De novo*** **acquisition of BCR-ABL1 mutations in (A) CML-T1 and (B) KCL-22 cells treated with PARP1 inhibitor NU-1025.** The presence of mutated mRNA for BCR-ABL1 was determined by NGS in cells treated (light columns) or untreated (dark columns) with 35 µM NU-1025 for CML-T1 and 40 µM NU-1025 for KCL-22, respectively, up to day 49 since the start of treatment. The growing concentration of imatinib is indicated above the columns.

Supplementary Figure S10

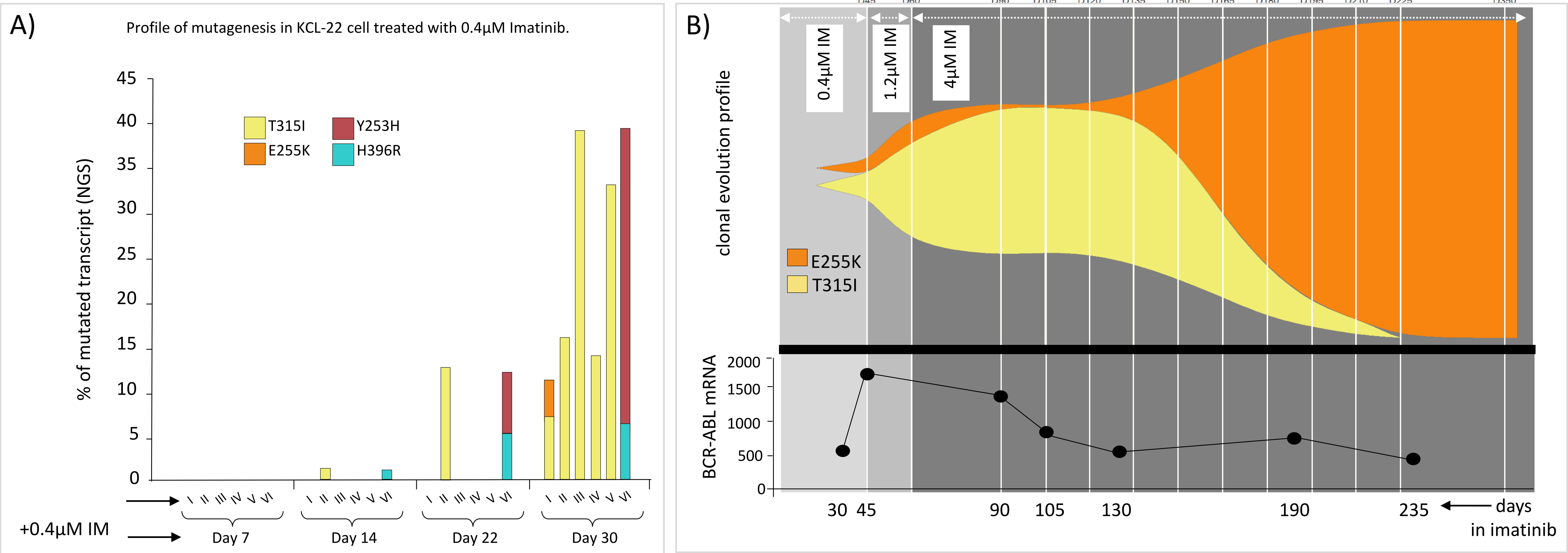

Supplementary Figure S10. The dynamics of BCR-ABL1 KD mutation expansion in KCL-22 cells depend on imatinib concentration and time of exposure. No BCR-ABL1 KD mutations were detected in *de novo* untreated KCL-22 cells. Mutated transcripts were detected after exposure of the cells to 0.4 µM imatinib in 6 independent experiments (i-vi). **(B)** BCR-ABL1 mutation profiles of KCL-22 cells after exposure to 0.4 µM imatinib followed by a subsequent increase in imatinib concentration up to 4 µM. BCR-ABL1 mRNA expression showed as BCR-ABL1/GUS (%) during the follow-up of imatinib-resistant KCL-22 cells. The fact that E255K-bearing cells overgrow T315I-bearing cells in KCL-22 culture *in vitro* may be explained by the acquisition of a second E255K mutation on the second Ph chromosome in the follow-up. **(C)** The dynamics of BCR-ABL1 mutation profiles of KCL-22 cells cultivated in 4 µM and reduced concentrations (2 µM; 1 µM) of imatinib in parallel for 90 days. BCR-ABL mutation profiles of KCL-22 cultures were determined by NGS at designated time points: The presence of mutated BCR-ABL1 transcripts was determined at selected time points up to day 235 by NGS and is shown as a percentage of total BCR-ABL1 transcripts in the cells.

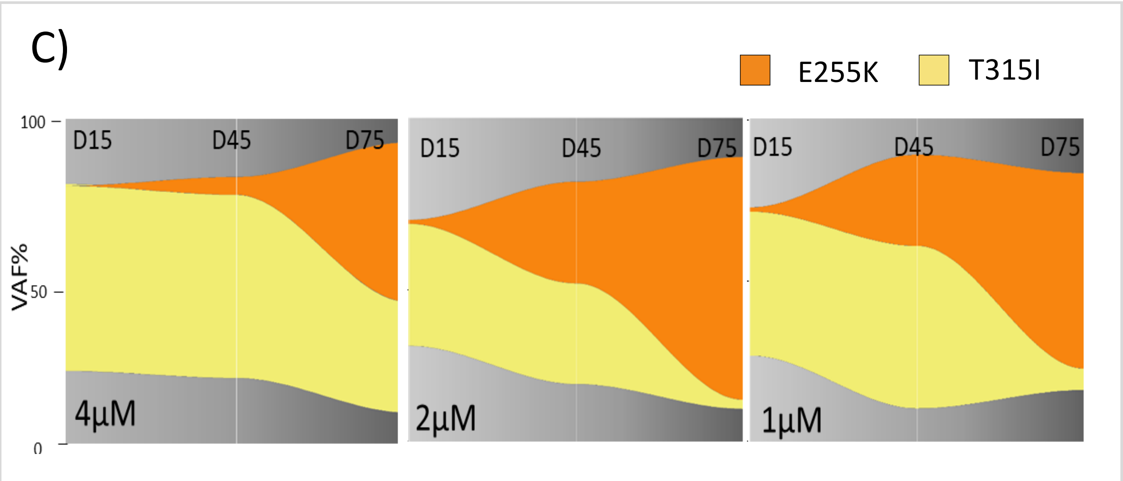

Supplementary Figure S11

A

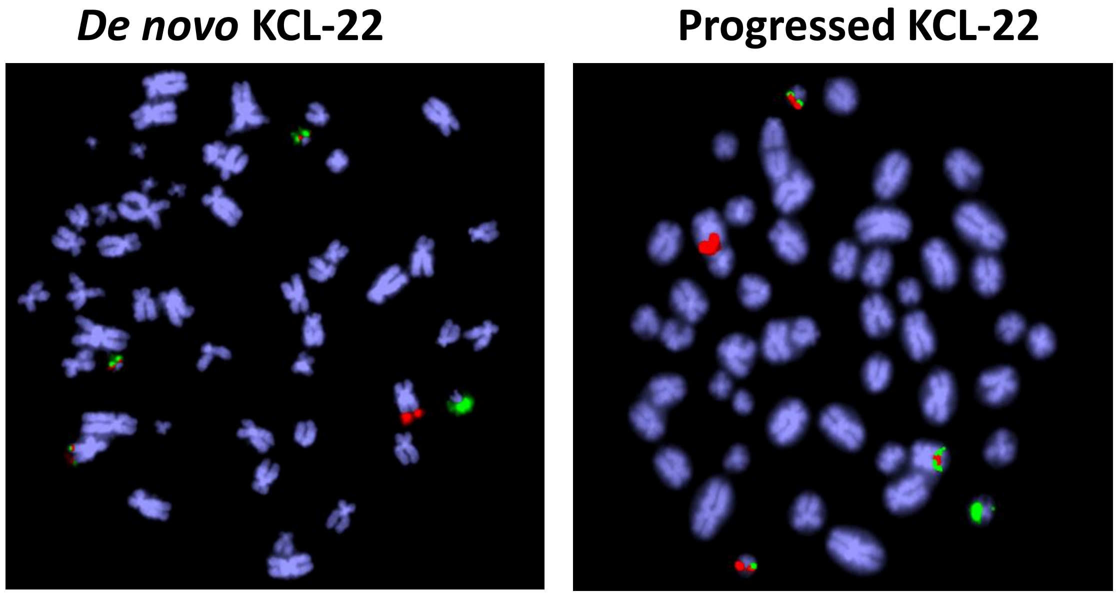

B

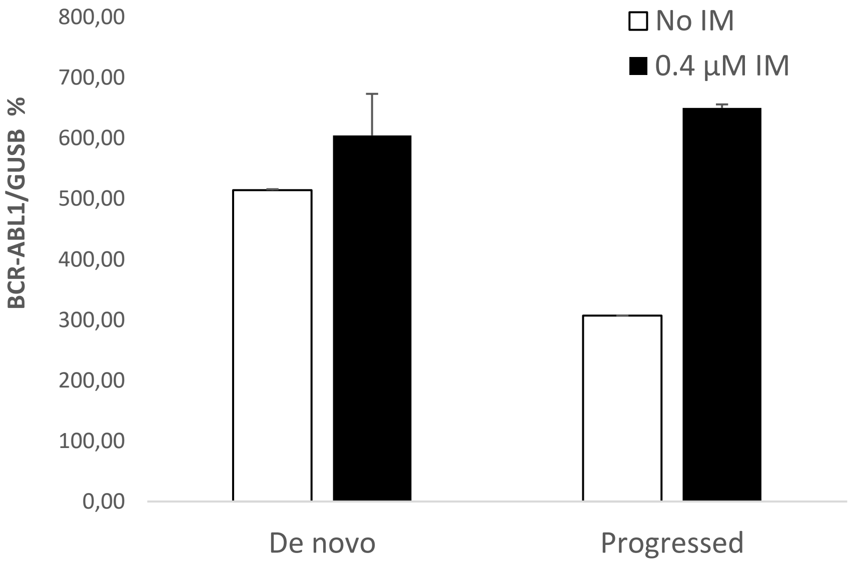

C

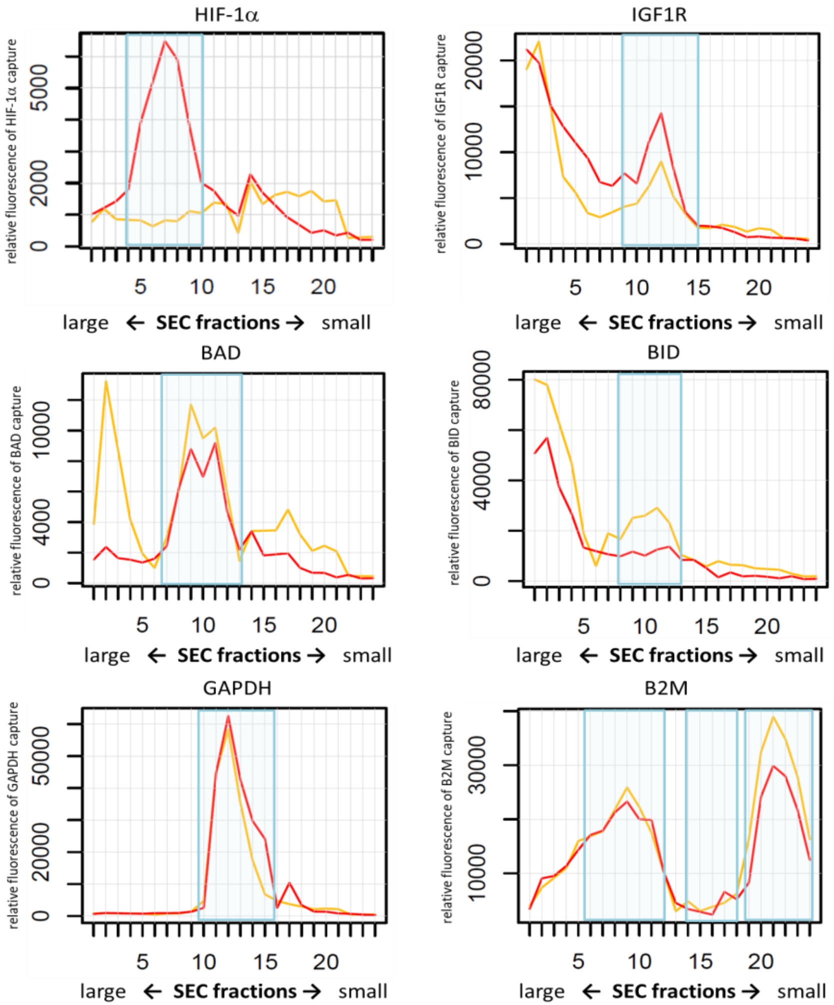

Supplementary Figure S11. Comparison of *de novo* (up to 30 passages postthawing) and progressed (imatinib-naïve long-term growing) KCL-22 cells. (A) FISH analysis did not reveal any difference in the karyotypes of *de novo* (left) and progressed (right) KCL-22 cells. **(B)** The expression of BCR-ABL1 mRNA in *de novo* (left) and progressed (right) KCL-22 cells. BCR-ABL1 expression in untreated (white columns) and imatinib-treated (black columns) cells was normalized to the expression of the GUSB control gene. **(C)** Levels of indicated proteins (protein entity defines within blue peak borders) in untreated (yellow line) and 0.4 µM imatinib treated (red line) progressed KCL-22 cells. GAPDH and β2-microglobulin (B2M) served as protein load controls. The proteomic data were obtained by SEC-MAP analysis and processed as described previously.[6]

Supplementary Figure S12

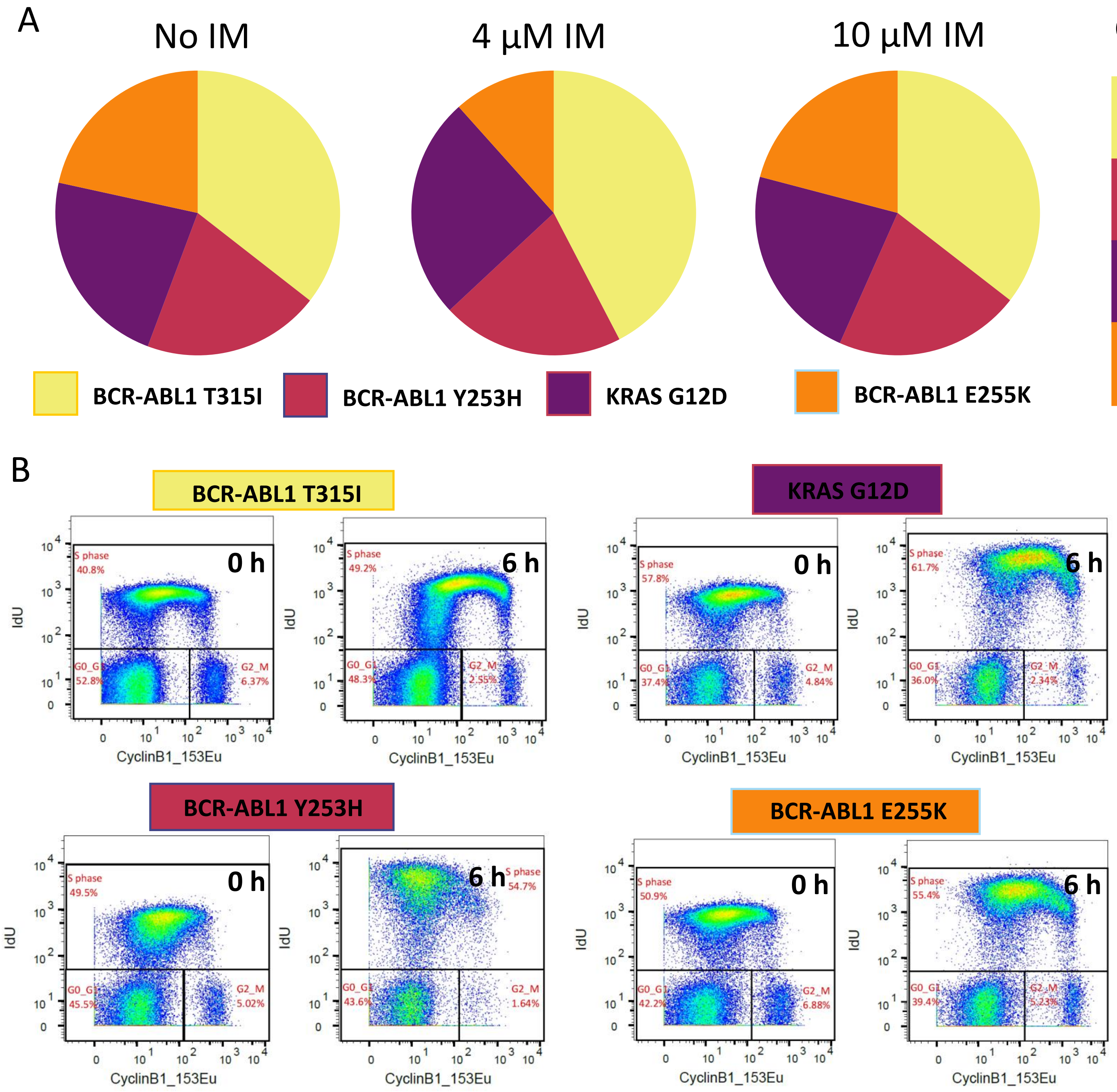

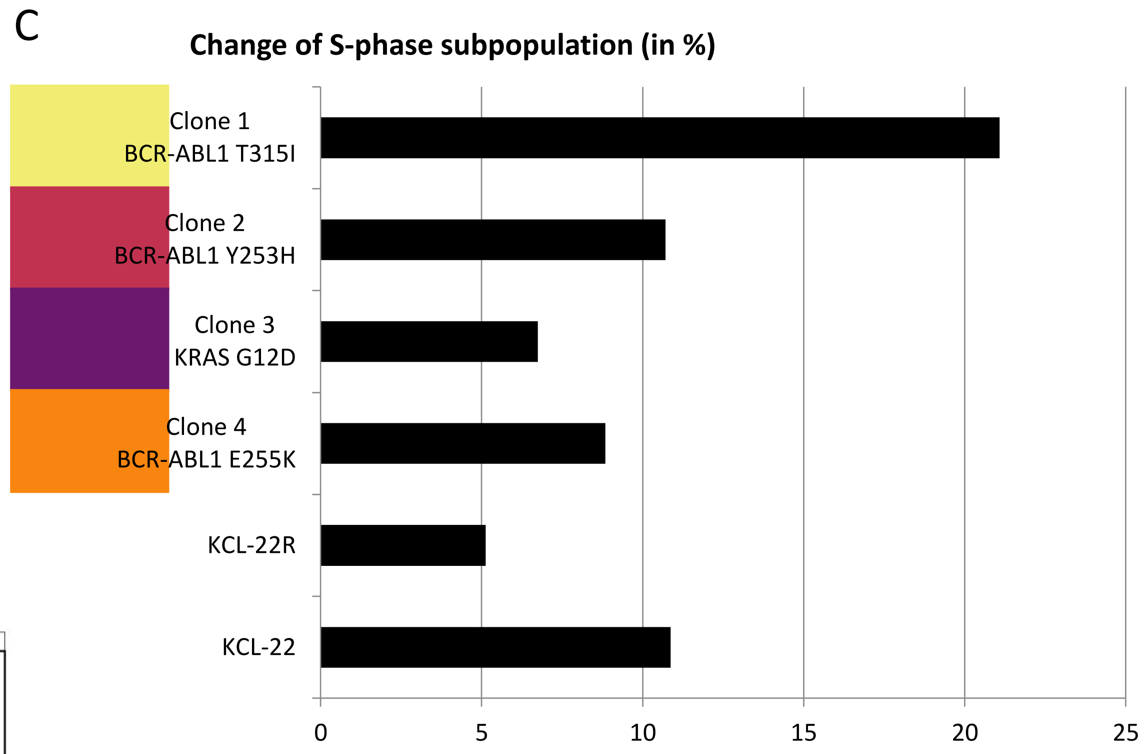

Supplementary Figure S12. Assessing the proliferation abilities of KCL-22R-resistant clones in different concentrations of imatinib. (A) Pie charts indicate the relative representation of individual KCL-22R clones in the mixture after 6 days of common growth in the medium without imatinib, with 4 μM imatinib or with 10 μM imatinib, starting from an equal number of cells ($1x10^6$) for each KCL-22R clone in the mixture. **(B)** Dot plots show relative proportion of cells for individual KCL-22R clones, growing in 4 μM imatinib, in different phases of cell cycle at the start point (0h) and 6 hours after the incubation of cells with 5-Iodo-2′-deoxyuridine (IdU). The changes of S phase subpopulation are indicated by red arrows with values expressed in percentage points. (**C**) The relative change in S-phase cell subpopulations in the KCL-22R clones growing in 4 μM imatinib was measured 6 hours after the incubation of cells with IdU.

Supplementary Figure S13

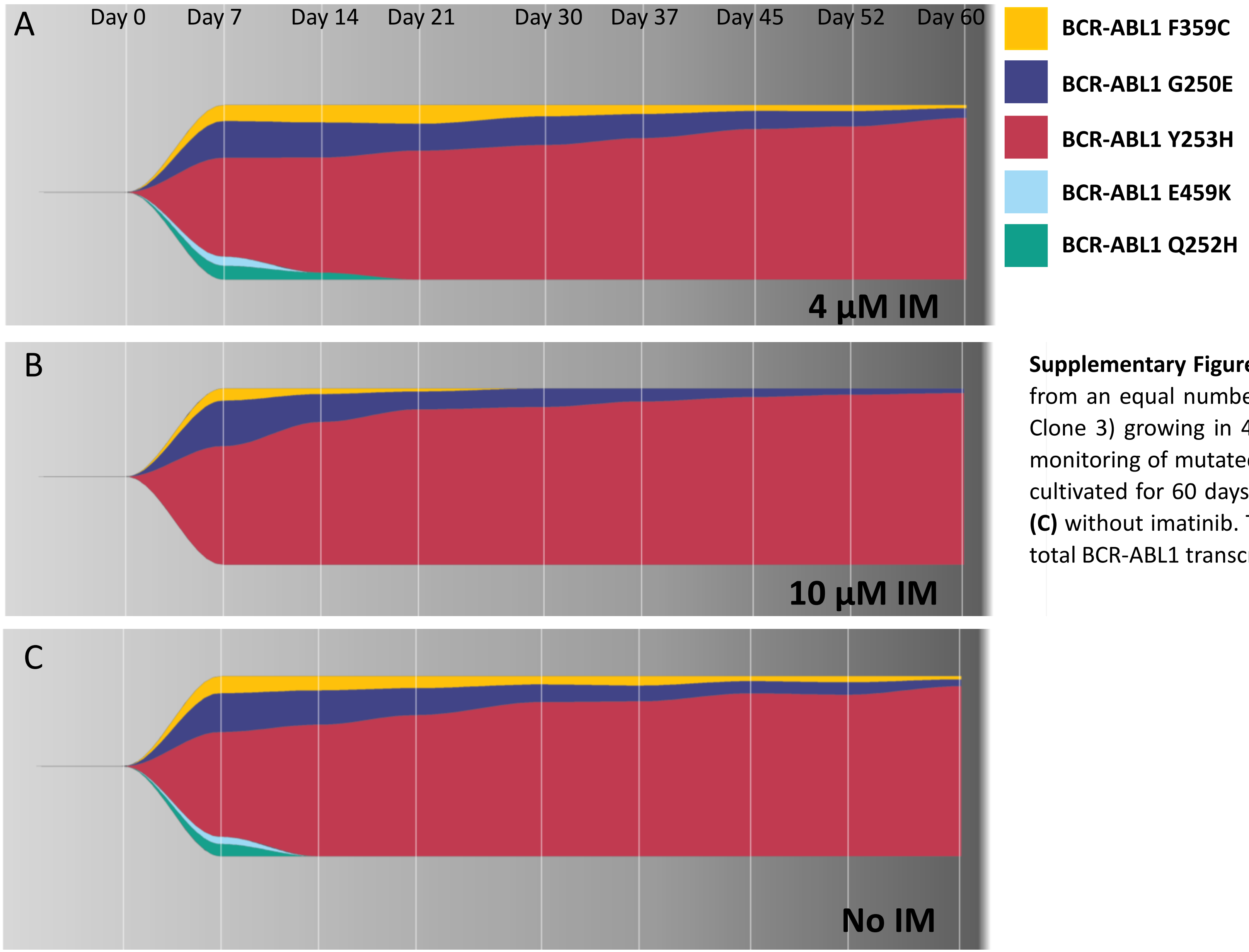

Supplementary Figure S13. The clonal evolution of CML-T1R-resistant clones. Starting from an equal number of cells ($1x10^6$) for each clone (CML-T1R Clone 1, Clone 2 and Clone 3) growing in 4 µM imatinib, subsequent clonal evolution was followed by the monitoring of mutated BCR-ABL1 transcripts by NGS at selected time points. Cells were cultivated for 60 days in stable conditions of **(A)** 4 µM imatinib, **(B)** 10 µM imatinib or **(C)** without imatinib. The presence of BCR-ABL1 mutations is shown as a percentage of total BCR-ABL1 transcripts in the cells.

Supplementary Figure S14

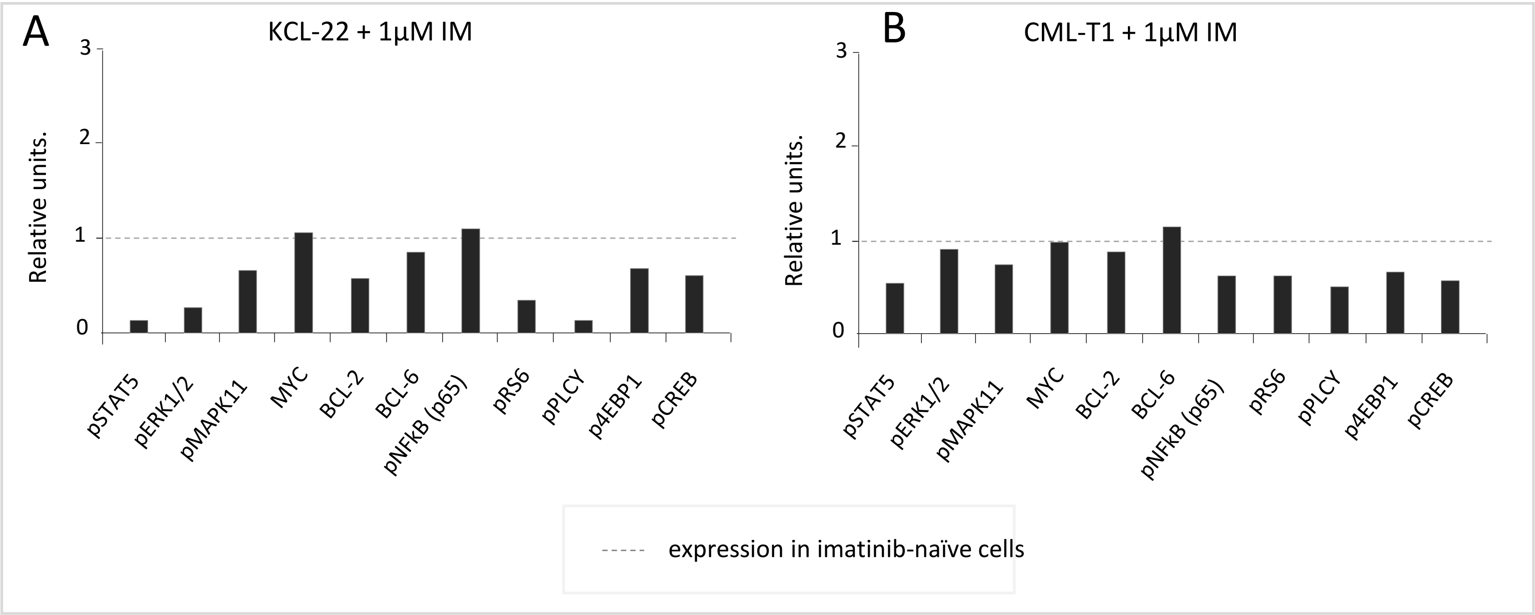

Supplementary Figure S14. The levels of phosphorylated (p) and unphosphorylated proteins determined by CyTOF in KCL-22 and CML-T1 cells. The dark columns represent the FC (relative expression) of protein expression for **(A)** KCL-22 and **(B)** CML-T1 cells exposed to 1 µM imatinib for 2 days. The FCs of protein levels in charts are related to the levels in imatinib-naïve KCL-22 and CML-T1 cells, respectively (marked by the dashed line with the level equalized to 1).

Supplementary Figure S15

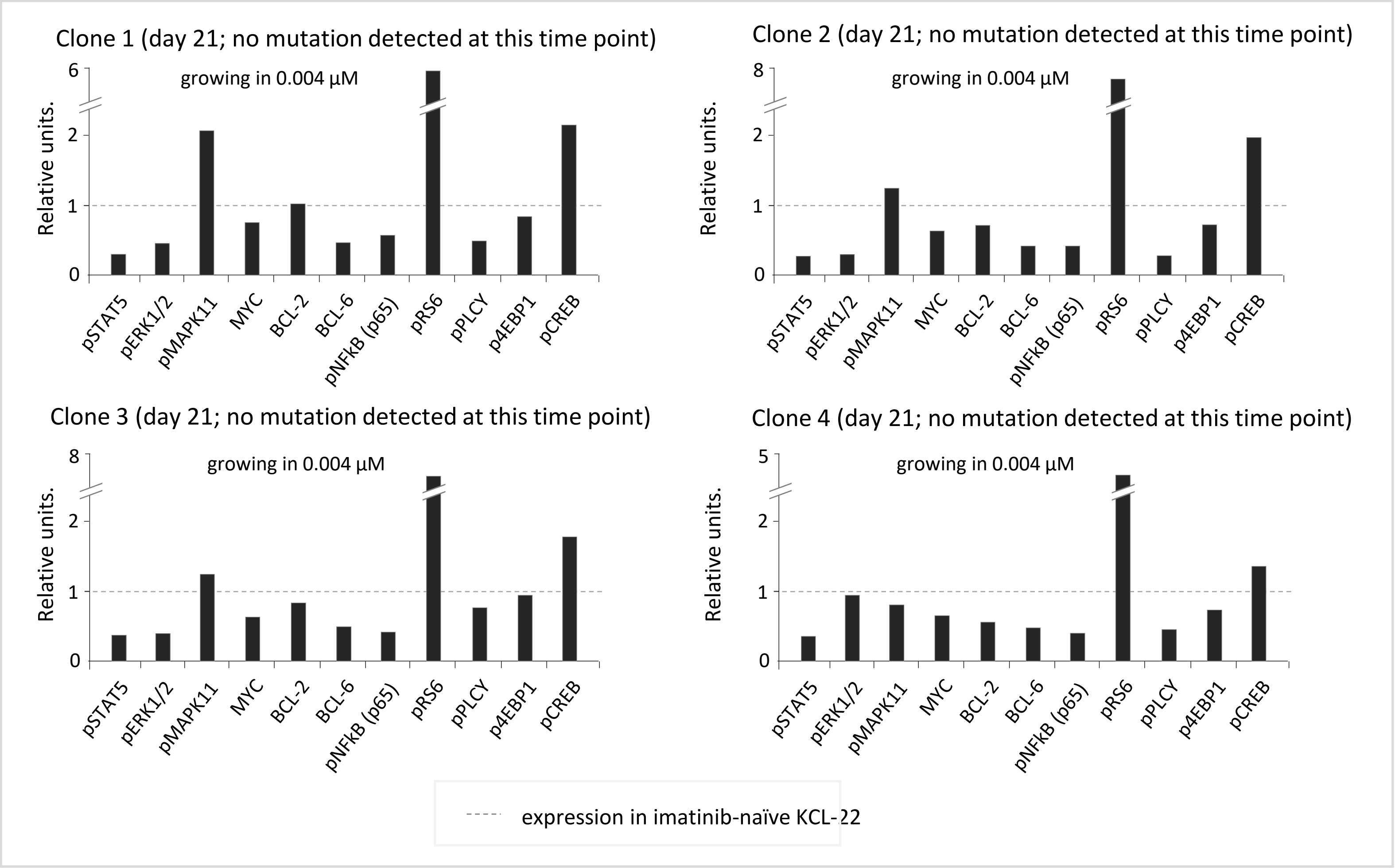

Supplementary Figure S15. The levels of phosphorylated (p) and unphosphorylated proteins determined by CyTOF in 4 KCL-22 clones at the early stage of resistance development. The black columns represent the FC (relative expression) of protein expression for 4 KCL-22 clones 21 days postsorting exposed to 0.004 µM imatinib (black columns). The FCs of protein levels in charts are related to the levels in imatinib-naïve KCL-22 cells (marked by the dashed line with the level equalized to 1).

Supplementary Figure S16

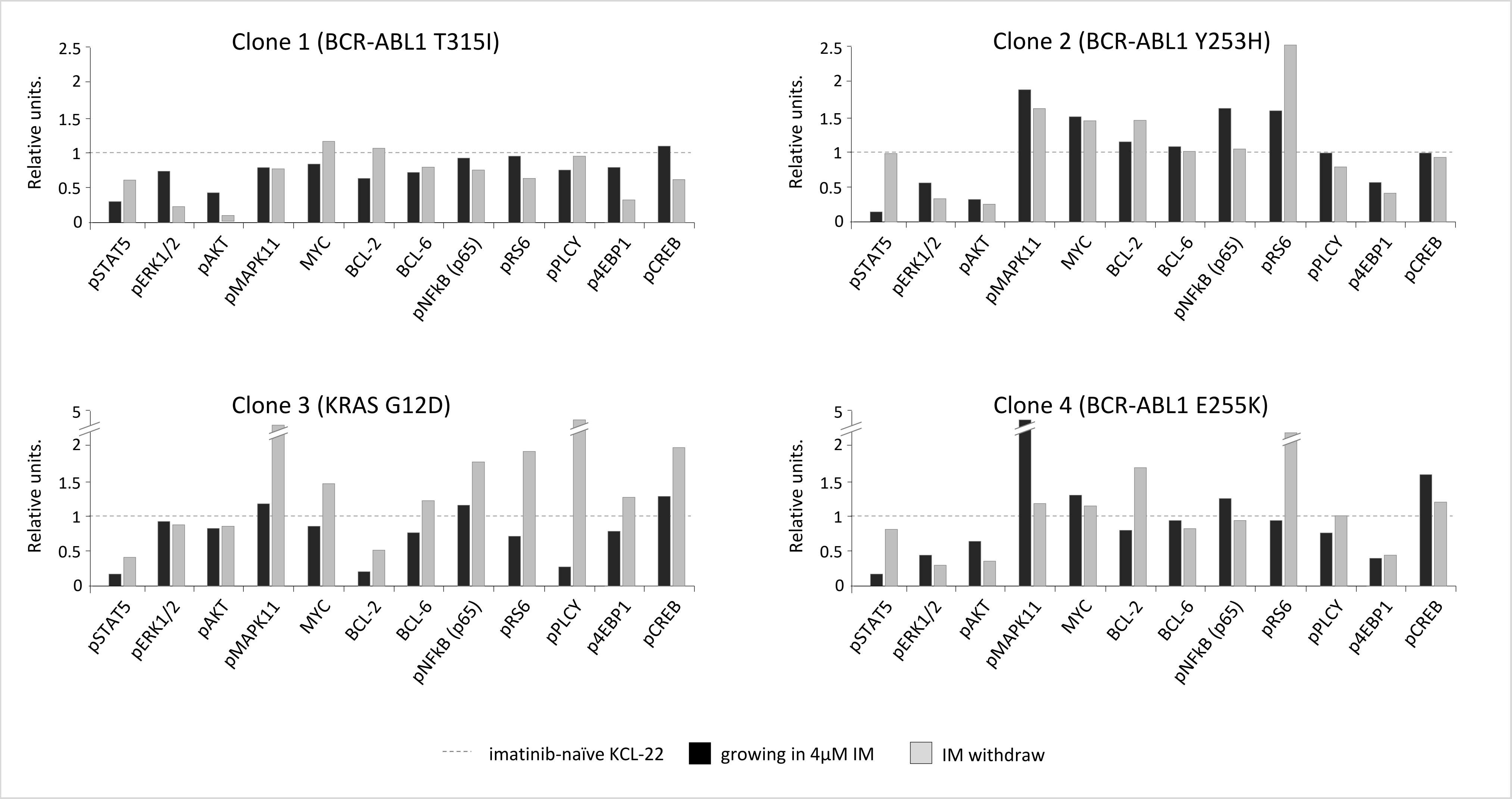

Supplementary Figure S16. The levels of phosphorylated (p) and unphosphorylated proteins determined by CyTOF in KCL-22R resistant clones. The columns represent the FC (relative expression) of protein expression for 4 KCL-22R clones (the type of mutation is indicated) growing in 4 µM imatinib (black columns) or in imatinib-free medium (imatinib withdraw for 1 week) (gray columns). The FCs of protein levels in charts are related to the levels in imatinib-naïve KCL-22 cells (marked by the dashed line with the level equalized to 1).

Supplementary Figure S17

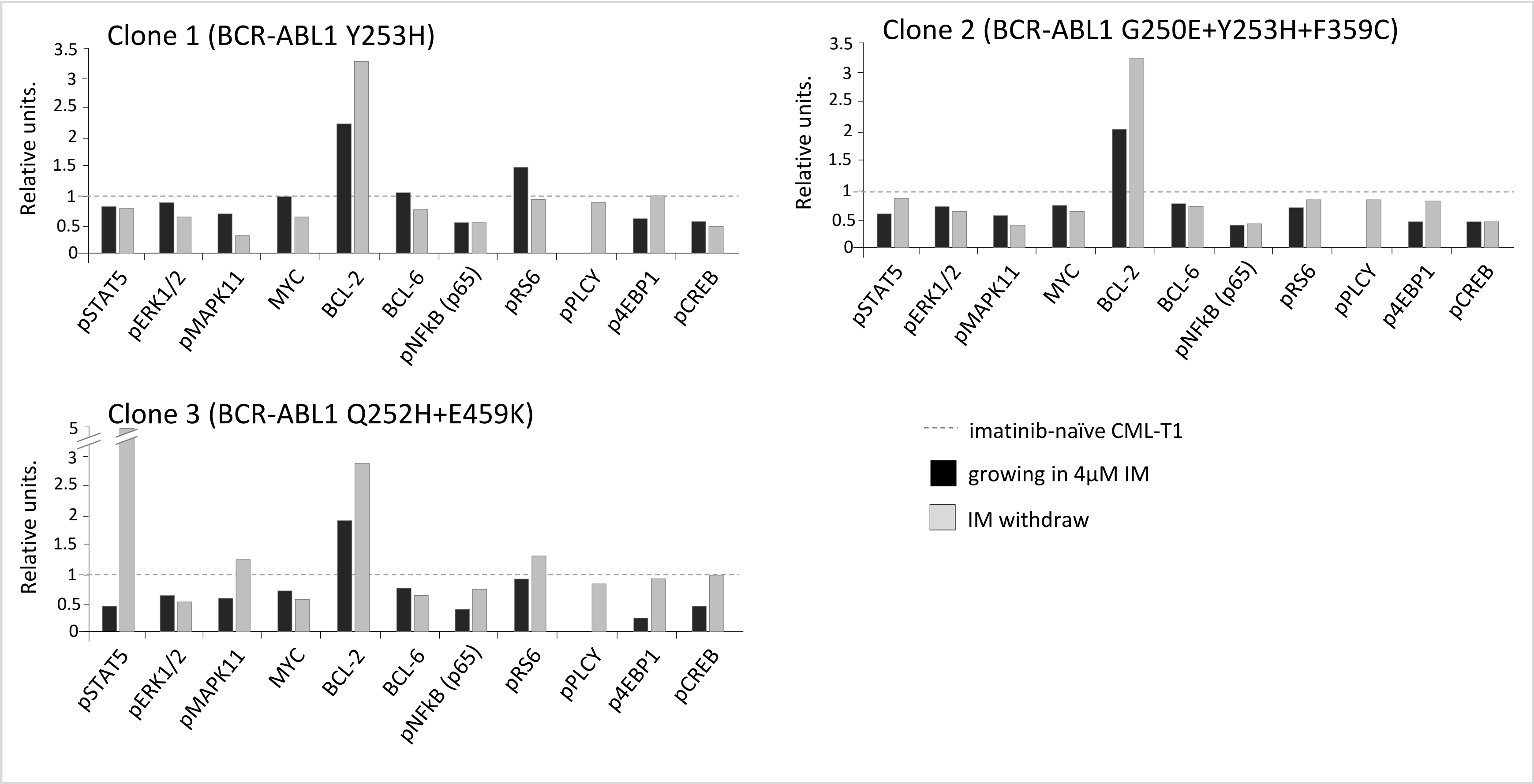

Supplementary Figure S17. The levels of phosphorylated (p) and unphosphorylated proteins determined by CyTOF in CML-T1R resistant clones. The columns represent the FC (relative expression) of protein expression for 4 CML-T1R clones (the types of mutation are indicated) growing in 4 µM imatinib (black columns) or in imatinib-free medium (imatinib withdraw for 1 week) (gray columns). The FCs of protein levels in charts are related to the levels in imatinib-naïve CML-T1 cells (marked by the dashed line with the level equalized to 1).

Supplementary Table S1: Individual genes in Human DNA Damage Signaling Pathway RT^2 Profiler™ PCR Array (PAHS-029Z; Qiagen) and Human DNA Repair RT^2 Profiler™ PCR Array (PAHS-042Z; Qiagen)

Symbol	Gene name
ABL1	C-abl oncogene 1, non-receptor tyrosine kinase
APEX1	APEX nuclease (multifunctional DNA repair enzyme) 1
APEX2	APEX nuclease (apurinic/apyrimidinic endonucleaze) 2
ATM	Ataxia telangiectasia mutated
ATR	Ataxia telangiectasia and Rad3 related
ATRIP	ATR interacting protein
ATRX	Alpha thalassemia/mental retardation syndrome X-linked
ATXN3	Ataxin 3
BARD1	BRCA1 associated RING domain 1
BAX	BCL2-associated X protein
BBC3	BCL2 binding component 3
BLM	Bloom syndrome, RecQ helicase-like
BRCA1	Breast cancer 1, early onset
BRCA2	Breast cancer 2, early onset
BRIP1	BRCA1 interacting protein C-terminal helicase 1
CCNH	Cyclin H
CCNO	Cyclin O
CDC25A	Cell division cycle 25 homolog A (S. pombe)
CDC25C	Cell division cycle 25 homolog C (S. pombe)
CDK7	Cyclin-dependent kinase 7
CDKN1A	Cyclin-dependent kinase inhibitor 1A (p21, Cip1)
CHEK1	CHK1 checkpoint homolog (S. pombe)
CHEK2	CHK2 checkpoint homolog (S. pombe)
CIB1	Calcium and integrin binding 1 (calmyrin)
CRY1	Cryptochrome 1 (photolyase-like)
CSNK2A2	Casein kinase 2, alpha prime polypeptide
DDB1	Damage-specific DNA binding protein 1, 127kDa
DDB2	Damage-specific DNA binding protein 2, 48kDa
DDIT3	DNA-damage-inducible transcript 3
DMC1	DMC1 dosage suppressor of mck1 homolog, meiosis-specific homologous recombination (yeast)
ERCC1	Excision repair cross-complementing rodent repair deficiency, complementation group 1 (includes overlapping antisense sequence)
ERCC2	Excision repair cross-complementing rodent repair deficiency, complementation group 2
ERCC3	Excision repair cross-complementing rodent repair deficiency, complementation group 3 (xeroderma pigmentosum group B complementing)
ERCC4	Excision repair cross-complementing rodent repair deficiency, complementation group 4
ERCC5	Excision repair cross-complementing rodent repair deficiency, complementation group 5
ERCC6	Excision repair cross-complementing rodent repair deficiency, complementation group 6
ERCC8	Excision repair cross-complementing rodent repair deficiency, complementation group 8
EXO1	Exonuclease 1
FANCA	Fanconi anemia, complementation group A
FANCD2	Fanconi anemia, complementation group D2
FANCG	Fanconi anemia, complementation group G

Symbol	Gene name
FEN1	Flap structure-specific endonuclease 1
GADD45A	Growth arrest and DNA-damage-inducible, alpha
GADD45G	Growth arrest and DNA-damage-inducible, gamma
H2AFX	H2A histone family, member X
HUS1	HUS1 checkpoint homolog (S. pombe)
LIG1	Ligase I, DNA, ATP-dependent
LIG3	Ligase III, DNA, ATP-dependent
LIG4	Ligase IV, DNA, ATP-dependent
MGMT	O-6-methylguanine-DNA methyltransferase
MAPK12	Mitogen-activated protein kinase 12
MBD4	Methyl-CpG binding domain protein 4
MCPH1	Microcephalin 1
MDC1	Mediator of DNA-damage checkpoint 1
MLH1	MutL homolog 1, colon cancer, nonpolyposis type 2 (E. coli)
MLH3	MutL homolog 3 (E. coli)
MMS19	MMS19 nucleotide excision repair homolog (S. cerevisiae)
MPG	N-methylpurine-DNA glycosylase
MRE11A	MRE11 meiotic recombination 11 homolog A (S. cerevisiae)
MSH2	MutS homolog 2, colon cancer, nonpolyposis type 1 (E. coli)
MSH3	MutS homolog 3 (E. coli)
MSH4	MutS homolog 4 (E. coli)
MSH5	MutS homolog 5 (E. coli)
MSH6	MutS homolog 6 (E. coli)
NBN	Nibrin
MUTYH	MutY homolog (E. coli)
NEIL1	Nei endonuclease VIII-like 1 (E. coli)
NEIL2	Nei endonuclease VIII-like 2 (E. coli)
NEIL3	Nei endonuclease VIII-like 3 (E. coli)
NTHL1	Nth endonuclease III-like 1 (E. coli)
OGG1	8-oxoguanine DNA glycosylase
PARP1	Poly (ADP-ribose) polymerase 1
PARP2	Poly (ADP-ribose) polymerase 2
PARP3	Poly (ADP-ribose) polymerase family, member 3
PCNA	Proliferating cell nuclear antigen
PMS1	PMS1 postmeiotic segregation increased 1 (S. cerevisiae)
PMS2	PMS2 postmeiotic segregation increased 2 (S. cerevisiae)
PNKP	Polynucleotide kinase 3'-phosphatase
POLB	Polymerase (DNA directed), beta
POLD3	Polymerase (DNA-directed), delta 3, accessory subunit
POLL	Polymerase (DNA directed), lambda
PPM1D	Protein phosphatase, Mg2+/Mn2+ dependent, 1D
PPP1R15A	Protein phosphatase 1, regulatory (inhibitor) subunit 15A
PRKDC	Protein kinase, DNA-activated, catalytic polypeptide
RAD1	RAD1 homolog (S. pombe)
RAD17	RAD17 homolog (S. pombe)
RAD18	RAD18 homolog (S. cerevisiae)
RAD21	RAD21 homolog (S. pombe)
RAD23A	RAD23 homolog A (S. cerevisiae)
RAD23B	RAD23 homolog B (S. cerevisiae)
RAD50	RAD50 homolog (S. cerevisiae)
RAD51	RAD51 homolog (S. cerevisiae)
RAD51B	RAD51 homolog B (S. cerevisiae)

**Symbol**	**Gene name**
RAD51C	RAD51 homolog C (S. cerevisiae)
RAD51D	RAD51 homolog D (S. cerevisiae)
RAD52	RAD52 homolog (S. cerevisiae)
RAD54L	RAD54-like (S. cerevisiae)
RFC1	Replication factor C (activator 1) 1, 145kDa
RAD9A	RAD9 homolog A (S. pombe)
RBBP8	Retinoblastoma binding protein 8
REV1	REV1 homolog (S. cerevisiae)
RNF168	Ring finger protein 168
RNF8	Ring finger protein 8
RPA1	Replication protein A1, 70kDa
RPA3	Replication protein A3, 14kDa
SIRT1	Sirtuin 1
SLK	STE20-like kinase
SMC1A	Structural maintenance of chromosomes 1A
SMUG1	Single-strand-selective monofunctional uracil-DNA glycosylase 1
SUMO1	SMT3 suppressor of mif two 3 homolog 1 (S. cerevisiae)
TDG	Thymine-DNA glycosylase
TOPBP1	Topoisomerase (DNA) II binding protein 1
TOP3A	Topoisomerase (DNA) III alpha
TOP3B	Topoisomerase (DNA) III beta
TP53	Tumor protein p53
TP53BP1	Tumor protein p53 binding protein 1
TP73	Tumor protein p73
TREX1	Three prime repair exonuclease 1
UNG	Uracil-DNA glycosylase
XAB2	XPA binding protein 2
XPA	Xeroderma pigmentosum, complementation group A
XPC	Xeroderma pigmentosum, complementation group C
XRCC1	X-ray repair complementing defective repair in Chinese hamster cells 1
XRCC2	X-ray repair complementing defective repair in Chinese hamster cells 2
XRCC4	X-ray repair complementing defective repair in Chinese hamster cells 4
XRCC5	X-ray repair complementing defective repair in Chinese hamster cells 5 (double-strand-break rejoining)
XRCC3	X-ray repair complementing defective repair in Chinese hamster cells 3
XRCC6	X-ray repair complementing defective repair in Chinese hamster cells 6
XRCC6BP1	XRCC6 binding protein 1
**CONTROL GENES**	
B2M	Beta-2-microglobulin
GAPDH	Glyceraldehyde-3-phosphate dehydrogenase
HPRT1	Hypoxanthine phosphoribosyltransferase 1
RPLP0	Ribosomal protein, large, P0

Supplementary Table S2. Genes and gene exons included in Custom NGS panel - SeqCap EZ HyperCap Nimble gen (Roche)

Gene symbol	Gene name	Exons
**ABL1**	Abelson murine leukemia viral oncogene homolog 1	4-10
**ASXL1**	Putative Polycomb group protein ASXL1	12
**ATRX**	ATP-dependent helicase ATRX, X-linked helicase II	8-10, 17-31
**BCOR**	BCL-6 Interacting Corepressor	All exons
**BCORL1**	BCL6 Corepressor Like 1	All exons
**CBL**	E3- ubiqutine protein ligase	8+9
**CEBPA**	CCAAT/enhancer-binding protein alpha	All exons
**DNMT3A**	DNA (cytosine-5)-methyltransferase 3A	All exons
**EZH2**	histone-lysine N-methyltransferase enzyme	All exons
**FLT3**	receptor-type tyrosine-protein kinase FLT3	14+15+20
**GATA1**	Erythroid transcription factor- GATA-binding factor 1	2
**GATA2**	GATA-binding factor 2	2-6
**IDH1**	Isocitrate dehydrogenase 1 (NADP+)- soluble	4
**IDH2**	Isocitrate dehydrogenase 2 (NADP+)- mitochondrial	4
**KIT**	Mast/stem cell growth factor receptor (SCFR)	2 + 8-19
**KRAS**	KRAS Proto-Oncogene, GTPase	2+3
**MLL (KMT2A)**	Histone-lysine N-methyltransferase 2A	5-11
**NPM1**	Nucleophosmin (NPM)	11
**NRAS**	N-ras protooncogene	2+3
**PHF6**	PHD finger protein 6	All exons
**PTEN**	Phosphatase and tensin homolog	5+7
**PTPN11**	Tyrosine-protein phosphatase non-receptor type 11	3+13
**RUNX1**	Runt-related transcription factor 1	All exons
**SF3B1**	Splicing factor 3B subunit 1	12-16
**SMC3**	Structural maintenance of chromosomes protein 3	10,13,19,23,25+28
**SRSF2**	Splicing factor, arginine/serine-rich 2	1
**TET2**	Tet methylcytosine dioxygenase 2	3-11
**TP53**	tumor suppressor p53	2-11
**U2AF1**	Splicing factor U2AF 35 kDa subunit	2+6
**WT1**	Wilms tumor protein	4-9
**ZRSR2**	U2 small nuclear ribonucleoprotein auxiliary factor 35 kDa subunit-related protein 2	All exons
**SIRT1**	NAD-dependent deacetylase sirtuin-1	All exons
**EZH1**	Histone-lysine N-methyltransferase EZH1	All exons
**PRPF8**	Pre-mRNA-processing-splicing factor 8	All exons

Supplementary Table S3: CyTOF antibodies

Target	Clone	Metal
pStat5[pY694]	47	150Nd
p-Erk1/2 (44/42)	D13.14.4E	171Yb
pAkt[S473]	D9E	163Dy
pMAPK11/p38 [180/182]	D3F9	156Gd
c-Myc	9E10	176Yb
BCL-2	Bcl-2/100	153Eu
BCL-6	K1-12-91	165Ho
pNF-kBp65[S536]	93H1	161Dy
pRS6[S240/244]	D68F8	159Tb
pPLCg2[Y759]	REA341	155Gd
p4E-BP1	236B4	149Sm
pCREB [S133]	87G3	165Ho
PARP (cleaved)	D64E10	143Nd
Caspase 3 (cleaved)	D175	170Er

Supplementary Table S4: The detection of somatic mutations in KCL-22R clones by NGS myeloid panel

Gene	Amino Acid Change	Mutation Call: Genomic	Position GRCh37.p13	Chromosome	COSMIC	dbSNP NCBI	Clone 1		Clone 2		Clone 3		Clone 4	
							D21	D205	D21	D205	D21	D205	D21	D205
ABL1	p.T315I	C>T	133748283	9	COSM12560	rs121913459		50						
ABL1	p.Y253H	T>C	133738357	9	COSM12576	rs121913461				30				
ABL1	p.E255K	G>A	133738363	9	COSM12573	rs121913448								50
KRAS	p.G12D	C>T	25398284	12	COSM521	rs121913529						50		
RUNX1	p.G74R	C>G	36259271	21		rs1435268371					34	35		
ATRX	p.K1933fs	delT	76855037	X	COSM6914754						26	42		
TP53	p.R306X	G>A	7577022	17	COSM10663	rs121913344							100	100
BCOR	p.R1454Q	C>T	39921459	X		rs1018691198				13				
DNMT3A	p.E228K	C>T	25471079	2	COSM2911841*		44	48	41	45	45	45	48	48
DNMT3A	p.V227L	C>A	25471082	2	COSM2911843*		43	48	41	45	45	44	47	48
PRPF8	p.R1402C	G>A	1564699	17	COSM2738826*	rs563667441	99	99	100	100	99	100	100	100
BCOR	p.V878A	A>G	39931966	X	COSM2964508*	rs759983308	100	100	100	100	100	100	100	100
TP53	p.P301Qfs	delG	7577036	17		rs876660726	91	93	92	93	91	88	94	100

*COSMIC Cell lines Project - known mutations in KCL-22 cells
